# Sarcospan protects against LGMD R5 via remodeling of the sarcoglycan complex composition in dystrophic mice

**DOI:** 10.1172/JCI187868

**Published:** 2025-06-19

**Authors:** Ekaterina I. Mokhonova, Daniel Helzer, Ravinder Malik, Hafsa Mamsa, Jackson Walker, Mark Maslanka, Tess S. Fleser, Mohammad H. Afsharinia, Shiheng Liu, Johan Holmberg, Z. Hong Zhou, Eric J. Deeds, Kirk C. Hansen, Elizabeth M. McNally, Rachelle H. Crosbie

**Affiliations:** 1 Department of Integrative Biology and Physiology, UCLA, Los Angeles, California, USA.; 2 Department of Biochemistry and Molecular Genetics, University of Colorado Anschutz Medical Campus, Aurora, Colorado, USA.; 3 Department of Microbiology, Immunology, and Molecular Genetics and; 4 California NanoSystems Institute, University of California, Los Angeles, California, USA.; 5 Department of Experimental Medical Science, Lund University, Lund, Sweden.; 6 Institute for Quantitative and Computational Biosciences, University of California, Los Angeles, California, USA.; 7 Center for Genetic Medicine, Northwestern University Feinberg School of Medicine, Chicago, Illinois, USA.; 8 Eli and Edythe Broad Center of Regenerative Medicine and Stem Cell Research,; 9 Molecular Biology Institute, and; 10 Department of Neurology, David Geffen School of Medicine, UCLA, Los Angeles, California, USA.

**Keywords:** Cell biology, Muscle biology, Skeletal muscle

## Abstract

The dystrophin-glycoprotein complex (DGC) is composed of peripheral and integral membrane proteins at the muscle cell membrane that link the extracellular matrix with the intracellular cytoskeleton. While it is well established that genetic mutations that disrupt the structural integrity of the DGC result in numerous muscular dystrophies, the 3D structure of the complex has remained elusive. Two recent elegant cryoEM structures of the DGC illuminate its molecular architecture and reveal the unique structural placement of sarcospan (SSPN) within the complex. SSPN, a 25 kDa tetraspanin-like protein, anchors β-dystroglycan to the β-, γ- and δ-sarcoglycan trimer, supporting the conclusions of biochemical studies that SSPN is a core element for DGC assembly and stabilization. Here, we advance these studies by revealing that SSPN provides scaffolding in δ-sarcoglycanopathies, enabling substitution of δ-sarcoglycan by its homolog, ζ-sarcoglycan, leading to the structural integrity of the DGC and prevention of limb-girdle muscular dystrophy R5. Three-dimensional modeling reveals that ζ-sarcoglycan preserves protein-protein interactions with the sarcospan, sarcoglycans, dystroglycan, and dystrophin. The structural integrity of the complex maintains myofiber attachment to the extracellular matrix and protects the cell membrane from contraction-induced damage. These findings demonstrate that sarcospan prevents limb-girdle muscular dystrophy R5 by remodeling of the sarcoglycan complex composition.

## Abstract 2

Transgenically expressed sarcospan selectively prevents gamma-sarcoglycanopathy by stabilizing a sarcoglycan subcomplex where zeta-sarcoglycan replaces gamma-sarcoglycan restoring membrane stability.

## Graphical abstract

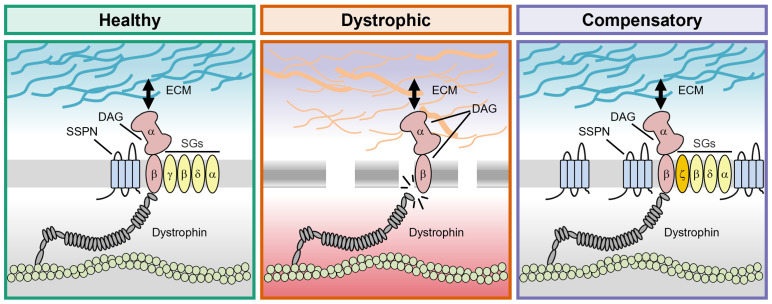

## Introduction

Muscular dystrophies encompass a spectrum of muscle wasting diseases characterized by progressive muscle weakness caused by myofiber damage and degeneration with subsequent fatty-fibrotic replacement of muscle tissue (1). Over 30 muscular dystrophies have been classified, with the most well studied being Duchenne muscular dystrophy (DMD) caused by mutations in the dystrophin gene (2, 3). Dystrophin is a core intracellular component of the dystrophin-glycoprotein complex (DGC) which links the intracellular cytoskeleton of skeletal muscle fibers to the extracellular matrix (ECM), conferring lateral force transmission, signal transduction, and sarcolemmal stability during muscle contraction (4–8). Loss of dystrophin impairs these functions, causing severe contraction-induced muscle injury (9, 10). As disease progresses, perpetual muscle fiber injury and loss of contractile tissue leads to whole-muscle weakness, respiratory impairment, cardiomyopathy, and premature death in the second or third decade of life (11).

The limb-girdle muscular dystrophies (LGMDs) primarily affect the muscles surrounding the scapular and pelvic girdles and result from various autosomal dominant and recessive pathogenic mutations in many genes (2, 12). Genetic mutations in any of the proteins in the sarcoglycan (SG) complex result in a subset of autosomal recessive LGMDs known as the sarcoglycanopathies (13, 14). The canonical SGs in skeletal muscle consist of three type II transmembrane glycoproteins (β-, γ-, and δ-SG) and one type I transmembrane glycoprotein (α-SG), with roles in DGC formation, ECM linkage, and mechanical signaling (15, 16). Mutations in the α- (*SGCA*), β- (*SGCB*), γ- (*SGCG*), and δ- (*SGCD*) SG genes cause the four sarcoglycanopathies termed LGMD R3, R4, R5, and R6, respectively (17). Two other SGs, ε- and ζ-SG, have been shown to produce alternative, low abundance SG complexes that are more enriched in nonmuscle cell and tissue types (18, 19). The sarcoglycanopathies are variable diseases initially described by their DMD-like pathology in patients (13, 20–24). Deficiency of any of the SGs typically result in secondary reduction of the entire subcomplex (25). The severity and biological effects of sarcoglycanopathy depend on precise mutations in sarcoglycans and varies between patients (25, 26). The diseased phenotypes have been recapitulated in both naturally occurring and genetically engineered animal models (13, 27–35). Animal model research reveals that gene therapy approaches to replace the defective sarcoglycan hold promise (36–38), and clinical studies are continually refining gene-based therapeutics (39, 40).

A commonality between several muscular dystrophies is membrane fragility caused by destabilization of the DGC (41, 42). Preclinical testing of synthetic membrane interacting molecules called poloxamers (43–45) and clinical trials of the steroid vamorolone (46–48) stabilize the sarcolemma. Additionally, skeletal muscle possesses redundant molecular mechanisms that partially or fully compensate for loss of gene function. For example, upregulation of cell-ECM adhesion complexes such as the utrophin-glycoprotein complex (UGC), in which dystrophin is replaced by its autosomal paralog utrophin, or the α7β1 integrin complex provides compensatory myofiber attachment (49, 50). In DMD patients and the *mdx* mouse model of DMD (51, 52), increased sarcolemmal abundance of the UGC and α7β1 integrin complexes partially compensated for the lack of dystrophin (53). Therefore, targeting these compensatory complexes offers valuable therapeutic potential.

Sarcospan (SSPN) is a 25 kDa transmembrane core component of the DGC and UGC (54) and forms a subcomplex with the SGs to stabilize α-dystroglycan to the cell surface. In addition, SSPN interacts with the α7β1 integrin complex (26, 54, 55). SSPN has been shown to relocalize to the membrane after gene therapy delivery of δ-SG in the δ-SG deficient hamster (55). SSPN expression at the muscle cell membrane is diminished as a consequence of complete or partial loss of the SGs in cases of LGMD (26) as well as multiple animal models (56, 57). Assembly of the SG-SSPN subcomplex remains to be completely understood but is thought to require initial association of β- and δ-SG followed by α- and γ-SG in the endoplasmic reticulum (58, 59). Interaction with SSPN likely occurs during transport to the membrane (60). There has been little investigation of the orthologous relationships within the sarcoglycan subcomplex of the DGC. Given the capacity for SSPN to rewire cell-matrix interactions in the context of DMD, we tested whether SSPN overexpression in murine models of sarcoglycanopathy (*Sgca*, *Sgcb*, and *Sgcg*) would affect muscle cell adhesion in the LGMD disease context. SSPN-transgenic expression in the sarcoglycanopathy models (*Sgca*^TG^, *Sgcb*^TG^, *Sgcg*^TG^) revealed that disease pathology was mitigated in the *Sgcg*, but not *Sgca* or *Sgcb*. Mechanistically, SSPN stabilized expression of a compensatory SG subcomplex in *Sgcg*^TG^ muscle that restored membrane stability. Enhancing SSPN expression can be used to stabilize the membrane and reduce disease severity in both dystrophin and γ-SG deficiency, demonstrating therapeutic potential for treating muscular dystrophies.

## Results

### SSPN ameliorates muscle histopathology in Sgcg skeletal muscle.

To investigate the effect of SSPN overexpression in the sarcoglycanopathies, we crossed α-, β-, and γ-SG–deficient mice with C57BL/6J WT SSPN transgenic mice in which the SSPN transgene is expressed under control of the human skeletal α-actin promotor (61), resulting in skeletal muscle–specific overexpression at threefold normal levels (Supplemental Figure 1; supplemental material available online with this article; https://doi.org/10.1172/JCI187868DS1).

**Figure 1:**
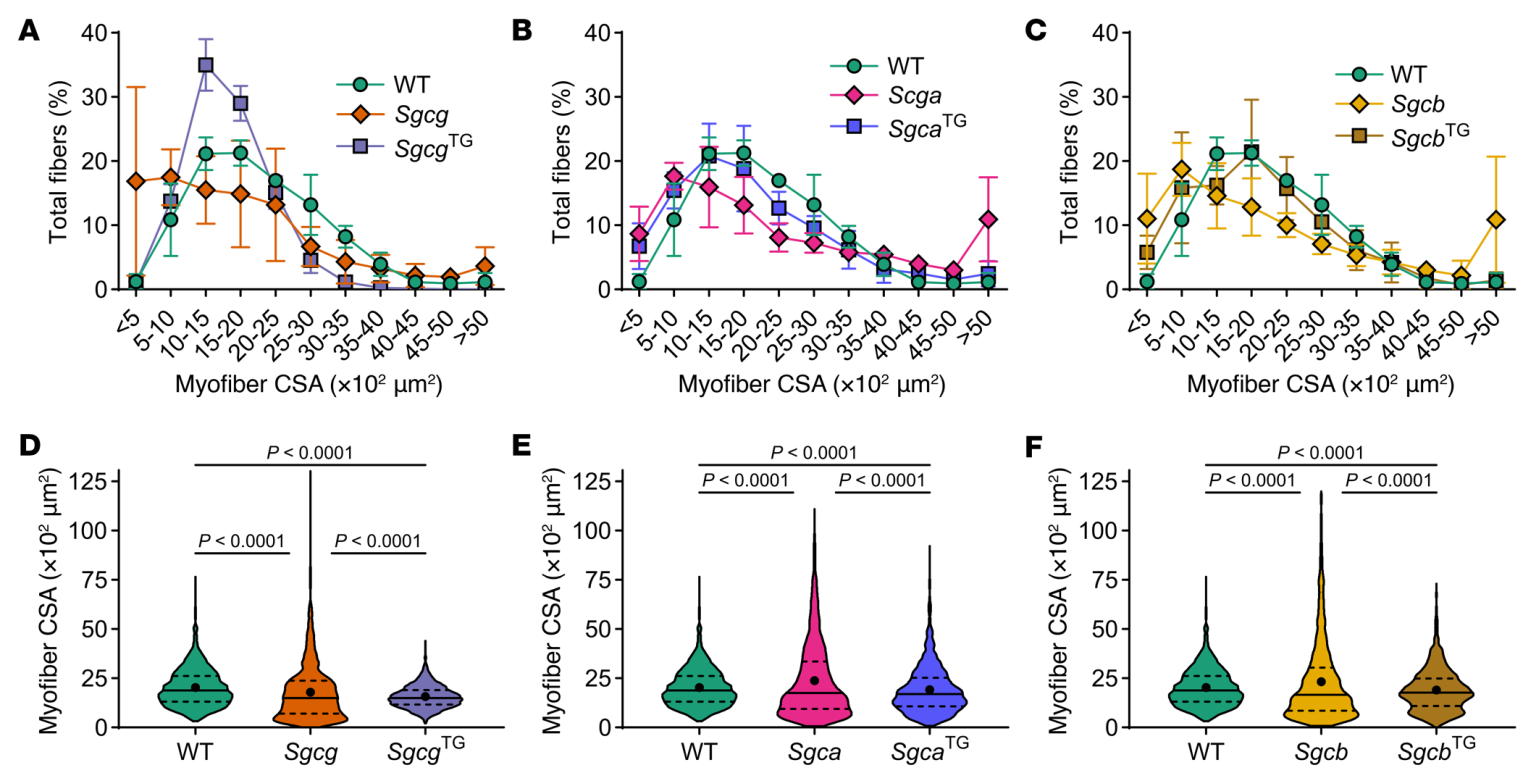
SSPN reduces fiber crosssectional area and fiber size variability. Crosssectional area analysis of quadriceps muscle fibers from g-sarcoglycan deficient (**A** and **D**), α-sarcoglycan–deficient (**B** and **E**) and β-sarcoglycan-deficient mice and (**C** and **F**) SSPN-transgenic littermates. Overexpression of SSPN reduced crosssectional area and fiber size variability across all three lines, most notable in γ-sarcoglycan–deficient muscle overexpressing SSPN. Data in (**D**–**F**) shows muscle fiber crosssectional area distributions (total 1,500 individual fibers), median (solid black line), mean (solid black circle), and first and third quartiles (dashed black line). Muscle fiber crosssectional area distributions were compared using pairwise Kolmogorov–Smirnov tests with Bonferroni correction. *n* = 3 mice per genotype, 500 fibers were measured per mouse.

**Figure 2:**
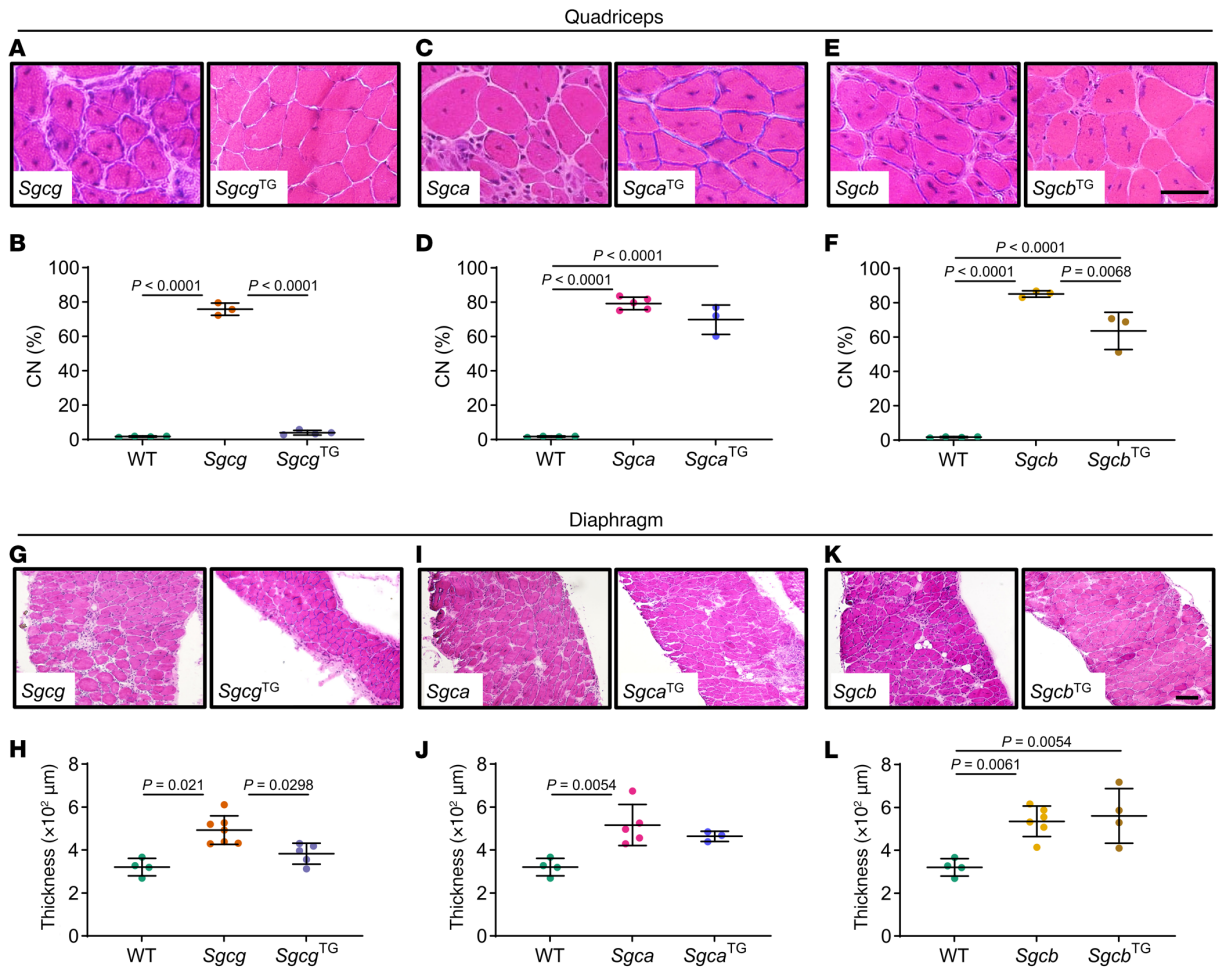
SSPN ameliorates central nucleation and reduces dystrophic pathology in γ-sarcoglycanopathy muscles. (**A**–**F**) Quantification of centrally located nuclei (CN, central nucleation) from H&E-stained quadriceps cryosections from (**A** and **B**) *Sgcg*, (**C** and **D**) *Sgca*, and (**E** and **F**) *Sgcb* mice and SSPN-transgenic littermates (*n* = 3–5 mice per genotype). The percentage of myofibers with centrally located nuclei was significantly increased in all 3 sarcoglycan-deficient lines. Overexpression of SSPN reduced central nucleation in *Sgcg* mice to WT levels, but not in *Sgca* or *Sgcb* mice. (**G**–**L**) Diaphragms from *Sgcg*^TG^ mice exhibit reduced dystrophic pathology. Low-magnification view of diaphragm muscle from (**G**) *Sgcg*, (**I**) *Sgca*, and (**K**) *Sgcb* mice and SSPN-transgenic littermates. The magnification is identical in all images. SSPN overexpression reduced diaphragm thickness in (**H**) *Sgcg* mice, but not in (**J**) *Sgca* or (**L**) *Sgcb* mice (*n* = 4–6 mice per genotype). Statistical analysis by 1-way ANOVA and Tukey’s test. Scale bars: 50 μm (**A**, **C**, and **E**); 200 μm (**G**, **I**, and **K**).

Features of LGMD include early onset skeletal muscle hypertrophy followed by muscle loss and cardiac impairment as disease progresses (62, 63), and many of these features are recapitulated in mouse models. Muscular dystrophy is characterized by ongoing degeneration alongside regeneration within the same muscle. Accordingly, we assessed muscle fiber cross section area (CSA) as a reflection of these pathological processes. *Sgca*, *Sgcb*, and *Sgcg* muscles exhibited a higher percentage of extremely large (> 5,000 μm^2^) and small (< 500 μm^2^) fibers than WT and *Sgca*^TG^, *Sgcb*^TG^, and *Sgcg*^TG^ (Figure 1, A–C). While mean fiber CSA did not drastically differ between genotypes (Figure 1, D–F), muscle fiber CSA distributions were significantly different between genotypes, with all transgenic muscles shifting toward smaller CSA. This outcome was most pronounced in *Sgcg*^TG^ quadriceps muscle (Figure 1D). Smaller myofiber CSA was associated with lower muscle mass in transgenic mice (Supplemental Figure 2C). Lower CSA variation suggested reduced injury and regeneration in transgenic muscles. Next, we performed H&E staining to assess central nucleation as an indicator of regeneration (64–66) and found an approximately 10% and approximately 20% reduction in central nucleation in *Sgca*^TG^ (Figure 2, C–D) and *Sgcb*^TG^ muscle (Figure 2, E and F) compared with *Sgca* and *Sgcb*, respectively. However, decreased central nucleation was not accompanied by improved membrane stability, reduction of collagen deposition, or increased muscle function in *Sgca*^TG^ or *Sgcb*^TG^ mice (Supplemental Figure 3). Reduced muscle mass and myofiber CSA is likely a result of transgenic SSPN expression, since transgenic WT mice exhibit a similar reduction in muscle mass and fiber CSA (Supplemental Figure 4). In contrast to the *Sgca*^TG^ and *Sgcb*^TG^, *Sgcg*^TG^ muscle exhibited very low levels of central nucleation, indistinguishable from WT (Figure 2, A and B), reflecting near complete prevention of muscle injury.

The sarcoglycanopathies exhibit mild to severe respiratory impairment (67, 68) and patients die from cardiac and respiratory failure. The diaphragm is the predominant inspiratory muscle and undergoes structural and functional changes in muscular dystrophy. Diaphragm fibrosis is a prominent feature in dystrophic mouse models and in human patients, and increased diaphragm thickness in mouse models reflects both fibrosis and hypertrophy (57, 69, 70). Reduced diaphragm thickness, indicative of correction to normal, was seen in *Sgcg*^TG^ mice compared with *Sgcg* (Figure 2, G and H). *Sgca* and *Sgcb* diaphragms were similar to transgenic counterparts (Figure 2, I–L), suggesting that both limb muscles and diaphragms are protected from injury specifically in the *Sgcg*^TG^ mice.

**Figure 3:**
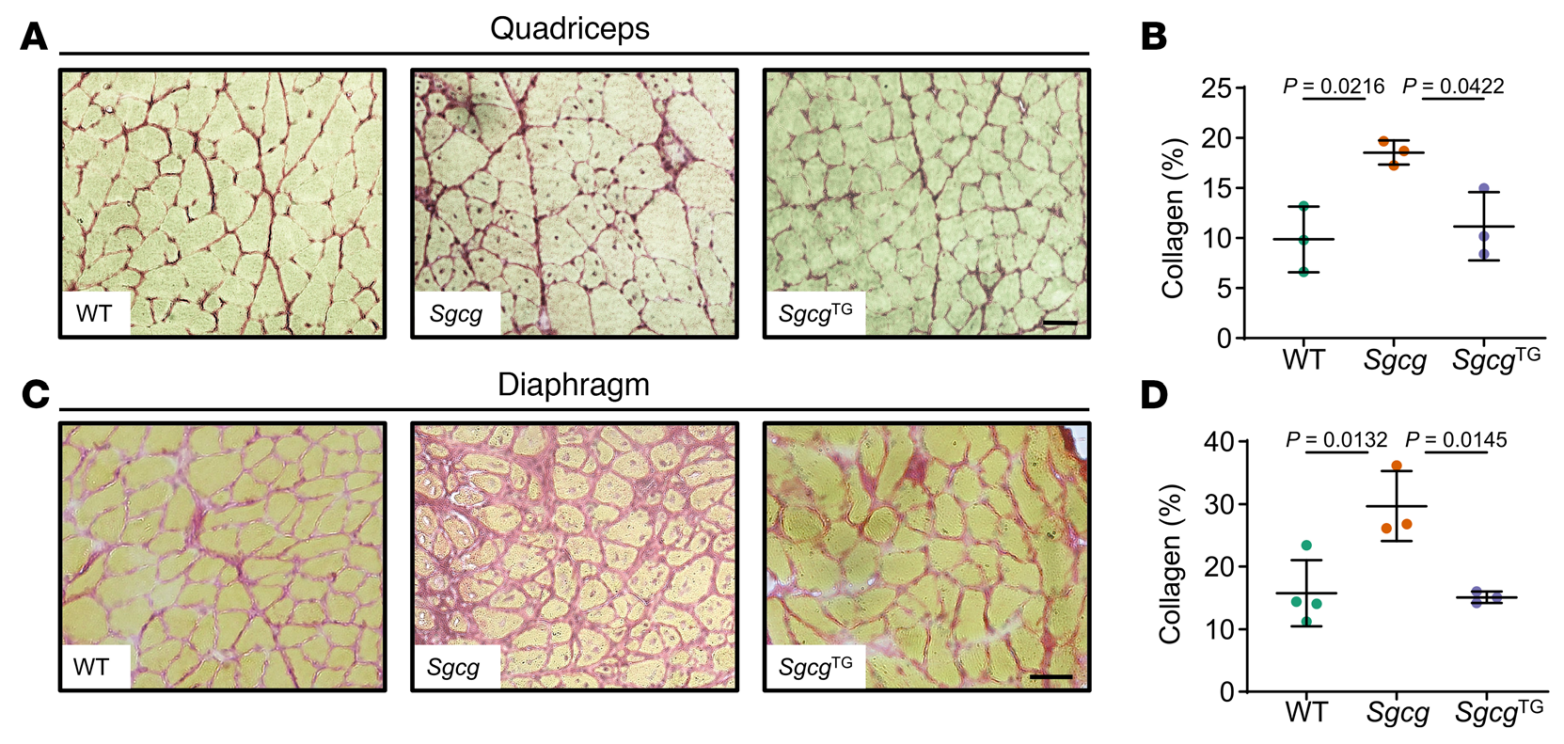
Fibrosis is prevented in *Sgcg*^TG^ muscle. Transverse cryosections and quantification of collagen area from (**A** and **B**) quadriceps and (**C** and **D**) diaphragm stained with Picrosirius Red. Overexpression of SSPN reduced collagen deposition throughout the diaphragm and quadriceps compared with *Sgcg*. *n* = 3–4 mice per genotype. Statistical analysis by 1-way ANOVA and Tukey’s test. Scale bars: 50 μm.

Given the data indicating amelioration of dystrophic hallmarks in the *Sgcg*^TG^ mice, we next assessed muscle fibrosis in the *Sgcg* and *Sgcg*^TG^ models. Picrosirius Red staining of 12-week-old muscles revealed increased collagen deposition in both the quadriceps (Figure 3, A and B) and diaphragm (Figure 3, C and D) of *Sgcg* mice and a reduction to WT levels in *Sgcg*^TG^ muscle. Together, these data reveal that SSPN prevents histopathological features in *Sgcg* muscle, but not *Sgca* or *Sgcb* muscle.

### SSPN restores membrane stability and after-exercise activity in Sgcg mice.

**Figure 4:**
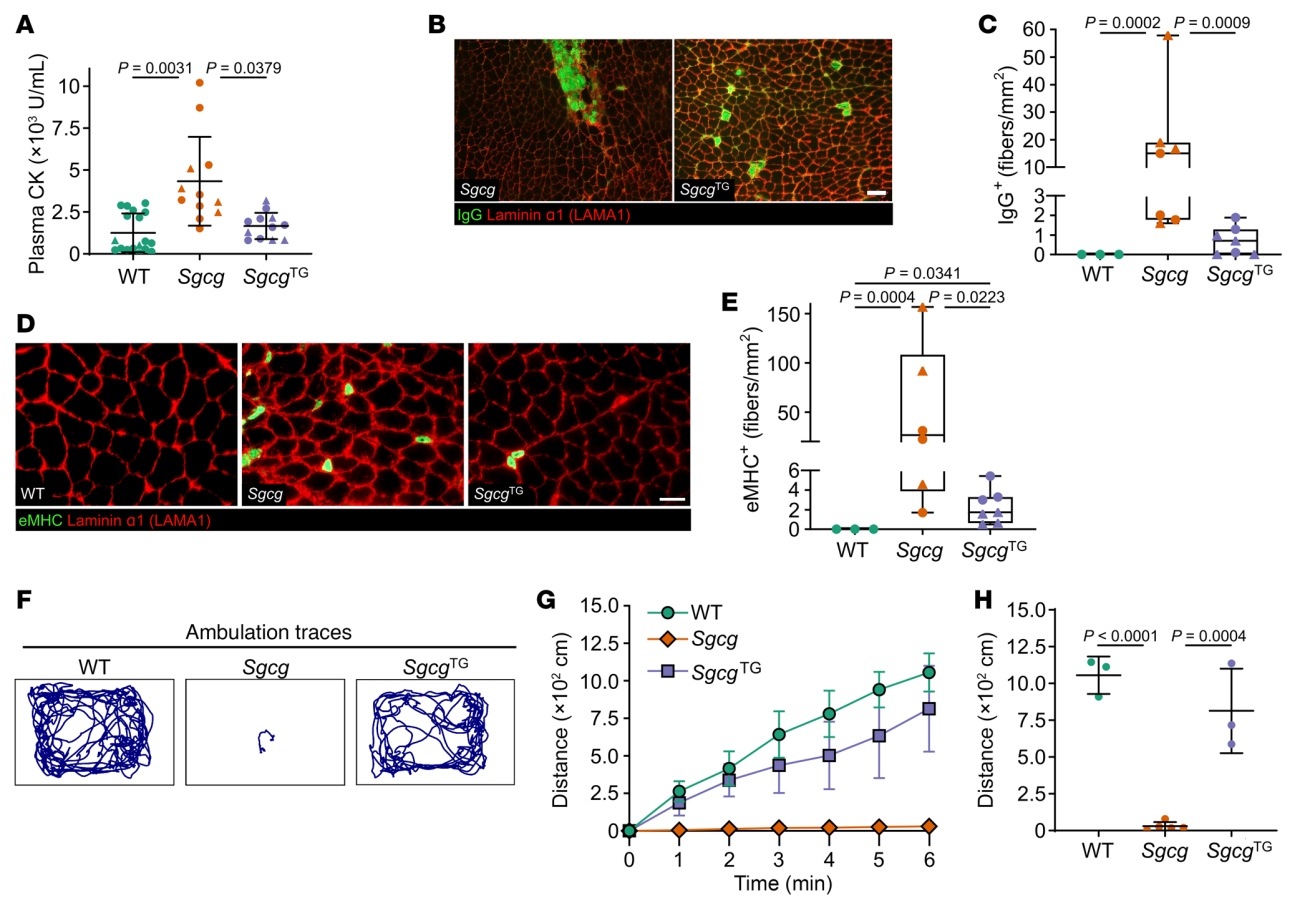
SSPN reduces membrane damage and improves muscle physiology in *Sgcg* mice. (**A**) Plasma creatine kinase (CK) levels of WT, *Sgcg*, and *Sgcg*^TG^ mice. Plasma CK in *Sgcg* mice was elevated approximately 4× that of WT mice. Overexpression of SSPN reduced CK back to WT levels. *n* = 12–18 per genotype (12–20 weeks of age). Statistical analysis by 1-way ANOVA and Tukey’s test. (**B**) Representative images of transverse sections of quadriceps muscles from 12-week-old *Sgcg* and *Sgcg*^TG^ mice stained with anti-mouse IgG antibody (green) and laminin (red). (**C**) IgG^+^ fibers counted from whole quadriceps images, *n* = 3–7. (**D**) eMHC (green) positive fibers were imaged as a measure of regeneration. Laminin (red) was used to outline fibers. (**E**) eMHC^+^ fibers were quantified and normalized to whole quadriceps image area. Both IgG^+^ and eMHC^+^ stains exhibited high variability across *Sgcg* mice, while *Sgcg*^TG^ muscle had very few positively stained fibers in both cases. Statistical analysis for IgG^+^ and eMHC^+^ fibers conducted by Kruskal-Wallis test and subsequent Conover-Iman test with Bonferroni correction. (**F**–**H**) Transgenic SSPN expression improved muscle physiology in *Sgcg* mice. (**F**) Representative traces of mouse ambulation in an open field during a 6-minute recording time. (**G** and **H**) Quantification of after-exercise activity distances from 30-week-old mice. *Sgcg*^TG^ mice traveled significantly farther distances compared with *Sgcg* littermates. *n* = 3–5 per genotype. Statistical analysis by 1-way ANOVA and Tukey’s test. Circles, male mice; triangles, female mice. Scale bars: 50 μm.

Membrane instability is the primary deficiency in sarcoglycanopathies, resulting from dysfunctional cytoskeleton to ECM connection. A clinical readout of sarcolemmal instability is elevated blood levels of muscle specific creatine kinase (CK), which is leaked from damaged myofibers, making it a common biomarker of muscle disease and injury (62, 71). Plasma CK concentration was elevated in all SG-deficient mice relative to WT mice (Figure 4A and Supplemental Figure 2D), with comparable levels between *Sgca*^TG^ and *Sgcb*^TG^ and nontransgenic counterparts. However, plasma CK in *Sgcg*^TG^ mice was reduced to WT levels (Figure 4A), revealing the membrane protective effects of SSPN in *Sgcg* muscle. We also directly assessed skeletal muscle tissue samples for indicators of membrane stability. Since muscle fiber injury and membrane damage result in an unregulated exchange of circulating factors such as IgG or IgM between the blood and the myofiber interior (72), the presence of intracellular IgG can be used as an alternative to the in vivo tracer Evan’s Blue dye assay (73). Immunofluorescence analysis revealed high variance of permeabilized myofibers across *Sgcg* samples and a greater number of IgG^+^ myofibers compared with WT (Figure 4, B and C). By contrast, *Sgcg*^TG^ muscle exhibited lower numbers of IgG^+^ myofibers compared with *Sgcg*. We next assessed newly regenerated myofibers using embryonic myosin heavy chain (eMHC), which similarly exhibited high variation in *Sgcg* muscle. There were significantly more eMHC^+^ myofibers in *Sgcg* muscle than WT muscle, which was corrected in *Sgcg*^TG^ muscle (Figure 4, D and E). Taken together, SSPN overexpression reduced membrane damage and elicited sarcolemmal stability in γ-sarcoglycanopathy.

Since SSPN overexpression prevented muscle injury, we next interrogated the effect of SSPN on muscle function. WT, *Sgcg*, and *Sgcg*^TG^ mice (30 weeks old) were subjected to a forelimb grip strength test followed by an after-exercise ambulation test. We performed 10 consecutive trials on each mouse 5 times and found that *Sgcg* mice were weaker than WT mice (data not shown). *Sgcg*^TG^ mice did not increase forelimb grip strength compared with nontransgenic littermates; however, they appeared to be more active than *Sgcg* littermates after exercise. Based on this observation, we conducted an open-field assay to test voluntary activity after grip strength exercise (74). Immediately following grip strength testing, mice were placed in a chamber and allowed to explore freely for 6 minutes, mimicking the clinically implemented 6-minute walk test used to assess disease status and progression (75). Mouse ambulation was video recorded to enable distance tracking over the testing period (Figure 4F). While *Sgcg* mice moved very little during the 6-minute after-exercise interval, the ambulatory distance of *Sgcg*^TG^ littermates was significantly increased throughout the trial, with a total walking distance similar to WT mice (Figure 4, G and H). This outcome may be attributed to SSPN-mediated treatment of fatigue-related aspects of dystrophic physiology (74).

### Dysregulated basement membrane composition in early stage Sgcg muscle is ameliorated by SSPN.

**Figure 5:**
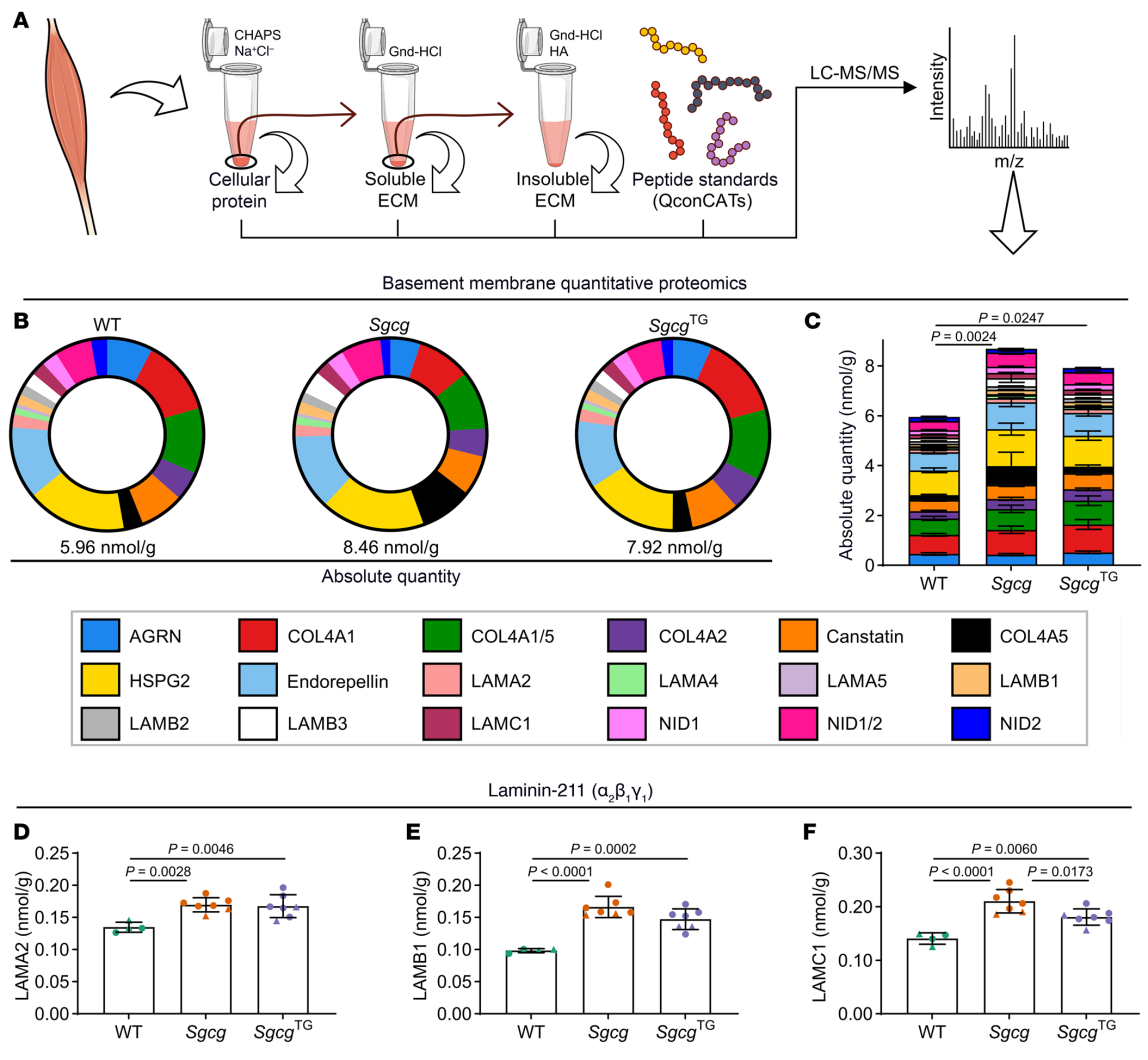
The basement membrane is expanded in *Sgcg* and *Sgcg*^TG^ muscle. Proteomics analysis was conducted using quantitative concatemers (QconCATs) to determine concentrations of basement membrane proteins. (**A**) Schematic overview of proteomics protocol designed to accurately capture difficult-to-extract ECM protein. The figure was partly generated using Servier Medical Art, provided by Servier and OpenStax, both licensed under Creative Commons Attribution 4.0 International. (**B**) Percentages of basement membrane proteins in WT, *Sgcg,* and *Sgcg*^TG^ muscle. (**C**) Comparison of total basement membrane protein content across the 3 genotypes. Basement membrane proteins were more abundant in both *Sgcg* and *Sgcg*^TG^ muscle compared with WT muscle. (**D**–**F**) Absolute quantity of LAMA2, LAMB1, and LAMC1 (protein components of laminin-211) from quantitative proteomics analysis. The laminin-211 proteins were elevated above WT in *Sgcg* and *Sgcg*^TG^ muscle. *n* = 4–7. Statistical analysis by 1-way ANOVA and Tukey’s test. Circles, male mice; triangles, female mice.

**Figure 6:**
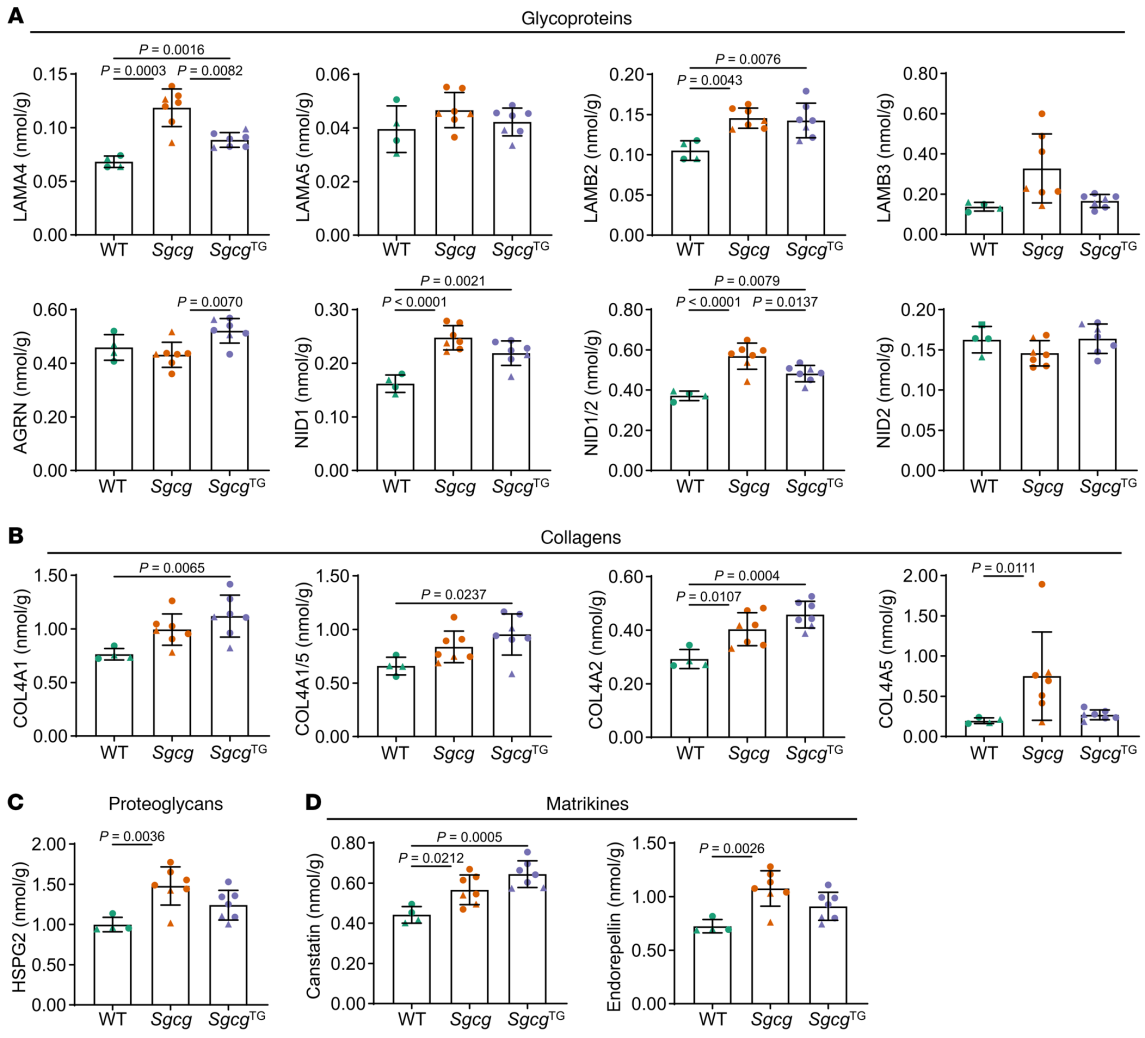
Distinct changes in basement membrane proteins in *Sgcg* and *Sgcg*^TG^ muscle. Quantitative proteomics plots of basement membrane proteins classified as (**A**) glycoproteins, (**B**) collagens, (**C**) proteoglycans, and (**D**) matrikines. The matrikines canstatin and endorepellin are peptide sequences found in the C-terminus of COL4A2 and HSPG2, respectively. NID1/2 represents the peptide sequence GNLYWTDWNR found in both NID1 and NID2. COL4A1/5 represents the peptide sequence SAPFIECHGR found in both COL4A1 and COL4A5. Type IV collagen was increased in *Sgcg*^TG^ samples, while the expanded basement membrane in *Sgcg* muscle was largely driven by regeneration-associated proteins HSPG2, endorepellin, NID1, and NID1/2. *n* = 4–7. COL4A5 and LAMB3 values were compared using Kruskal-Wallis test followed by Dunn’s test. LAMA4 values were compared using Welch’s ANOVA and Dunnett test. All other protein comparisons were conducted by 1-way ANOVA and Tukey’s test. Circles, male mice; triangles, female mice.

In skeletal muscle, the extracellular matrix is composed of 2 layers: the interstitial matrix and the basement membrane. We have shown that the interstitial matrix, which is mainly comprised of type I and type III collagens (76), is expanded in *Sgcg* muscle (Figure 3). The basement membrane directly interacts with receptors α-/β-dystroglycan and a7b1 integrins, localized at the myofiber sarcolemma. We sought to determine how loss of the SG-SSPN subcomplex in *Sgcg* muscle affected deposition of the basement membrane at an early stage of disease (8–12 weeks old). For these studies, we employed a sequential extraction method that enabled assessment of protein solubility by calculating the distribution of protein signals identified in the soluble and insoluble ECM fractions (77–79) (Figure 5A). These methods reveal the importance of using a wide range of detergents and chaotropic agents for extensive ECM digestion, enabling more complete protein recovery. We employed an absolute quantitative proteomics approach where protein fractions prepared for LC-MS/MS were combined with stable isotope–labeled quantitative concatemers (QconCATs) representing over 80 ECM and ECM-associated proteins (80, 81). We found that the basement membrane was expanded in both *Sgcg* and *Sgcg*^TG^ muscle relative to WT muscle (Figure 5, B and C) with notable distinctions between the models. In diseased *Sgcg* muscle, increased HSPG2, endorepellin (matrikine generated from C-terminus of HSPG2), and NID1 likely reflect a state of injury and regeneration (82, 83) and account for a large proportion of the expanded basement membrane in *Sgcg* muscle (Figure 5, B and C, and Figure 6, A–D). In *Sgcg*^TG^ muscle, there was an increase in the absolute quantity and proportion of type IV collagen (Figure 5B, Figure 6B, and Supplemental Tables 1 and 2). Type IV collagen provides an overall scaffolding function and binds various basement membrane proteins (83), which may be a unique feature of *Sgcg*^TG^ basement membrane. Several laminins, including LAMA2, LAMB1, and LAMC1 (constituents of the most abundant laminin trimer in skeletal muscle, laminin-211 [refs. 84, 85]), were elevated in both *Sgcg* and *Sgcg*^TG^ muscle (Figure 5, D–F, and Figure 6A) despite reduced injury and regeneration in *Sgcg*^TG^. Basement membrane proteins in *Sgcg* muscle are likely products of myogenic lineage cells (e.g., activated satellite cells and newly regenerated myofibers) and not ECM-producing cells derived from fibroadipogenic progenitors, as these cells downregulate several basement membrane genes (Supplemental Figure 5). Dysregulated basement membrane composition in *Sgcg* muscle may result from a perpetual state of myogenesis (86). Increased basement membrane deposition in *Sgcg*^TG^ muscle, however, is not a result of regeneration and is not evidently controlled at the transcriptional level (Supplemental Figure 5), suggesting protein level regulation.

### Presence and glycosylation of α-dystroglycan and its laminin binding capacity is unaffected in Sgcg muscle.

**Figure 7:**
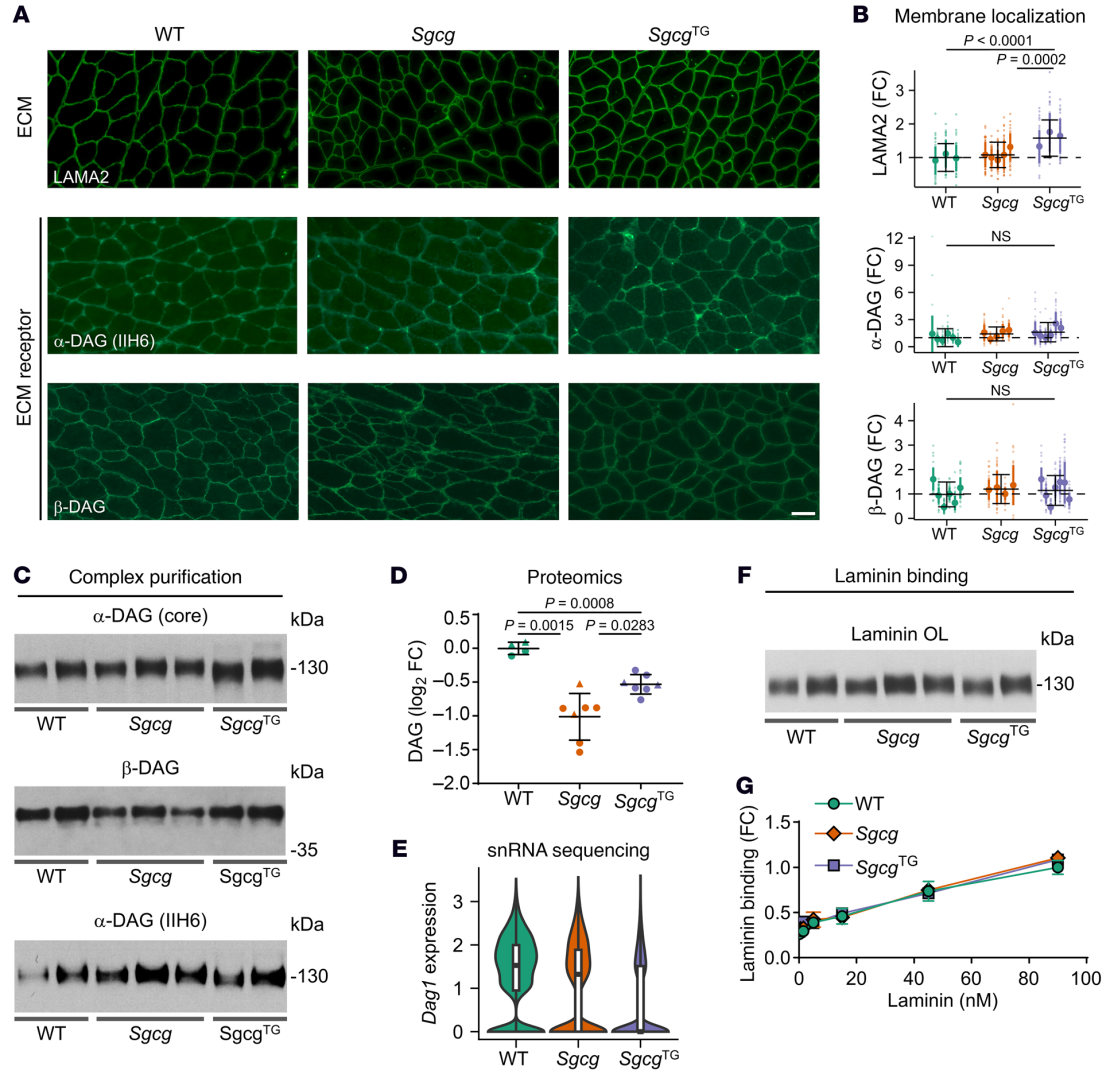
SSPN does not affect the presence of ECM receptors at the membrane or laminin binding capacity in *Sgcg* muscle. (**A**) Immunofluorescence assay quadriceps cryosections probed for laminin and dystroglycan (DAG) with (**B**) quantification. Scale bar: 50 mm; *n* = 3–6 mice per genotype, 49 individual myofiber measurements per mouse (total 147–294). Data are presented as FC relative to WT (mean ± SD) for individual mice within a genotype. The mean ± SD for each genotype is provided with black solid bars (dashed line represents mean of WT samples). Statistical analysis by fitting a generalized linear model. (**C**) Immunoblotting of eluate fractions of lectin (sWGA) purifications using the indicated antibodies (*n* = 2–3). IIH6 detects the laminin binding glycoepitope of α-DAG. (**D**) Proteomics analysis quadriceps muscle, *n* = 4–7. Data presented as log_2_(FC) relative to WT. Statistical analysis by unpaired *t* test and Benjamini-Hochberg procedure. (**E**) *Dag1* expression in myonuclei from single nuclei RNA-seq of quadriceps muscles. (**F**) Laminin overlays of sWGA-enriched eluates. (**G**) Laminin binding capacity of sWGA-enriched muscle lysates by solid phase laminin binding assay, *n* = 2–3. Circles, male mice; triangles, female mice.

Dystroglycan is translated as a single polypeptide that is posttranslationally processed to produce α- and β-subunits that span the membrane to connect dystrophin, localized to the intracellular face of the membrane, with the extracellular matrix (87). α-dystroglycan is heavily glycosylated with the matriglycan carbohydrate moiety that directly interacts with perlecan, laminin, and agrin matrix proteins to mediate cell adhesion (87–89). While it has been documented that laminin, β-dystroglycan, and dystrophin are normally expressed at the membrane in γ-sarcoglycan–deficient muscle (31), neither the integrity of the DGC nor its laminin-binding function have been investigated. Given our findings of increased laminin in *Sgcg* and *Sgcg*^TG^ muscle, we first probed the localization of laminin and dystroglycan by indirect immunofluorescence analysis of transverse muscle cryosections from all genotypes and found that SSPN increased laminin levels at the *Sgcg*^TG^ sarcolemma relative to WT and *Sgcg* muscle, while membrane dystroglycan levels were indistinguishable between the 3 genotypes (Figure 7, A and B). Laminin and dystroglycan were also localized to the membrane in both *Sgca* and *Sgca*^TG^ but reduced in *Sgcb* muscle (Supplemental Figure 6A). Next, we performed succinylated wheat germ agglutinin (sWGA) enrichment of skeletal muscle lysates from WT, *Sgcg*, and *Sgcg*^TG^ muscles. The sWGA lectin specifically binds glycoproteins with GlcNAc modifications (90), which are abundant in the DGC (primarily α-dystroglycan), making this enrichment useful for isolating dystroglycan and its associated proteins. Consistent with their membrane localization, analysis of sWGA enrichments revealed robust isolation of α- and β-dystroglycan from all samples (Figure 7C). We also detected glycosylated α-dystroglycan using IIH6 antibodies that bind to the matriglycan epitope, which comprises the major site for laminin binding (4). α-dystroglycan levels were similar between *Sgcg* and *Sgcg*^TG^ eluates (Figure 7C) while dystroglycan abundance in whole muscle lysate and mRNA expression were reduced in *Sgcg* and *Sgcg*^TG^ compared with WT (Figure 7, D and E). These data suggest that α-dystroglycan is properly glycosylated to support laminin interaction. To directly assess whether the enriched α-dystroglycan possesses different laminin binding capacity between genotypes, we performed 2 laminin binding assays. In a laminin binding overlay, purified laminin-111 was bound to denatured sWGA eluates immobilized by SDS-PAGE. In a solid phase laminin binding assay, purified laminin-111 was bound to sWGA enrichments immobilized on microtiter plate (91). Both the laminin binding overlay and solid phase laminin binding assay support that *Sgcg* muscle retains WT levels of laminin binding, even in the absence of the SG-SSPN subcomplex (Figure 7, F and G). This is likely a result of retained α-dystroglycan–laminin binding capacity and may also reflect integrin-laminin binding (Supplemental Figure 7). Additionally, these results revealed that laminin-binding capacity is preserved and not negatively impacted by SSPN in *Sgcg*^TG^ samples (Figure 7, F and G).

### SSPN restores sarcoglycan membrane localization in Sgcg muscle.

**Figure 8:**
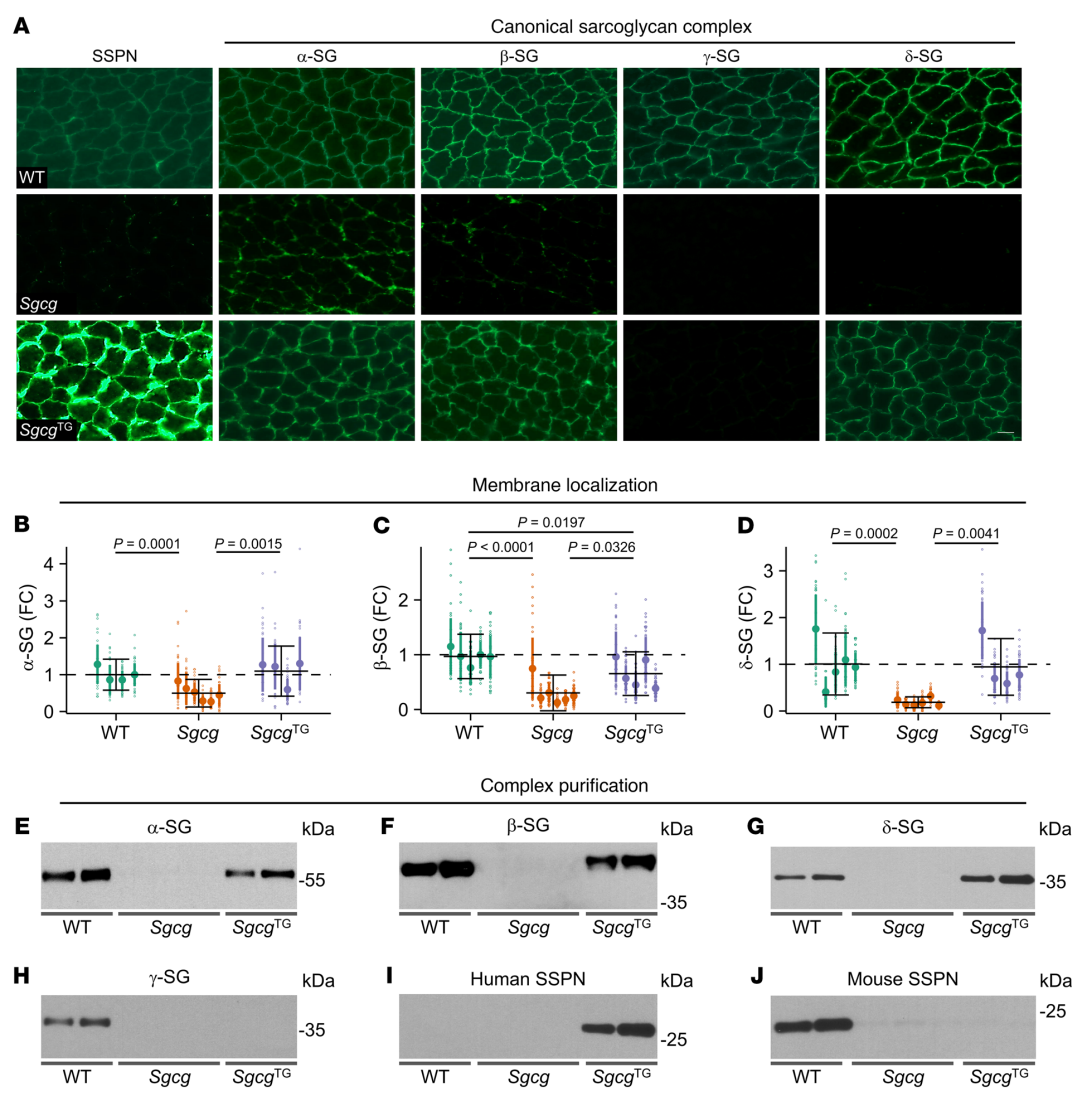
SSPN restores membrane expression protein interactions of α-, β- and δ-sarcoglycans. (**A**) Indirect immunofluorescence assays of transverse WT, *Sgcg*, and *Sgcg*^TG^ quadriceps cryosections using the indicated antibodies. Scale bar: 50 μm. (**B**–**D**) Quantification of sarcoglycan abundance at the sarcolemma, *n* = 4–6 mice per genotype, 49 individual myofiber measurements per mouse (total 196–294 myofibers per genotype). Data are presented as fold change (FC) relative to WT (mean ± SD) for individual mice within a genotype. The mean ± SD for each genotype is provided with black solid bars (dashed line represents mean of WT samples). Statistical analysis was performed by fitting a generalized linear model. (**E**–**J**) Immunoblotting of the eluate fractions of lectin (sWGA) purifications from all 3 genotypes using the indicated antibodies (*n* = 2–3 per genotype). Proteins that interact in a complex can be detected in eluate fractions.

Since dystroglycan membrane association and laminin binding were not responsible for amelioration of pathology in *Sgcg*^TG^ muscle, we next assessed membrane localization of DGC components by immunofluorescence staining. The SGs were absent or reduced in SG-deficient models, with a slight retention of α- and β-SG in *Sgcg* muscle, consistent with previously published results (32) (Figure 8A and Supplemental Figure 6B). SSPN abundance was substantially increased in all transgenic models (Figure 8A and Supplemental Figure 6B). Interestingly, α-, β-, and δ-SG were present at the membrane of *Sgcg*^TG^ muscle despite the absence of γ-SG, while SG were not membrane localized in *Sgca*^TG^ or *Sgcb*^TG^ muscle (Figure 8A and Supplemental Figure 6B). Fluorescence intensity quantification revealed that α-, β-, and δ-SG intensities return to or approach WT levels in *Sgcg*^TG^ muscle (Figure 8, B–D). Semiquantitative proteomics analysis of WT, *Sgcg*, *Sgcg*^TG^ quadriceps revealed that protein levels of α-, β-, and δ-SG were reduced in *Sgcg* muscle relative to WT while SG levels in *Sgcg*^TG^ muscle were increased above *Sgcg* (Supplemental Figure 8, A–C). Differences in SG abundance and membrane localization occurred, despite similar mRNA expression levels between genotypes (Supplemental Figure 8, D–I).

We further analyzed sWGA-enriched muscle lysates as an approach to interrogate assembly of the DGC complex (54, 91). Immunoblotting of sWGA enrichments showed that α-, β-, γ-, and δ-SG were reduced or absent in *Sgcg* samples (Figure 8, E–H). However, we found a restoration of α-, and β-, and δ-SG in *Sgcg*^TG^ samples, in accordance with immunostaining. hSSPN was abundant in *Sgcg*^TG^ samples, whereas mouse SSPN was reduced in *Sgcg* and *Sgcg*^TG^ samples (Figure 8, I and J). Reduced mouse SSPN in *Sgcg* sWGA fractions reflects DGC destabilization, whereas reduction in *Sgcg*^TG^ may result from regulatory downregulation by transgenic hSSPN (61). Together, these experiments revealed that SSPN overexpression restored membrane localization and association of α-, β-, and δ-SG in the absence of γ-SG.

**Figure 9:**
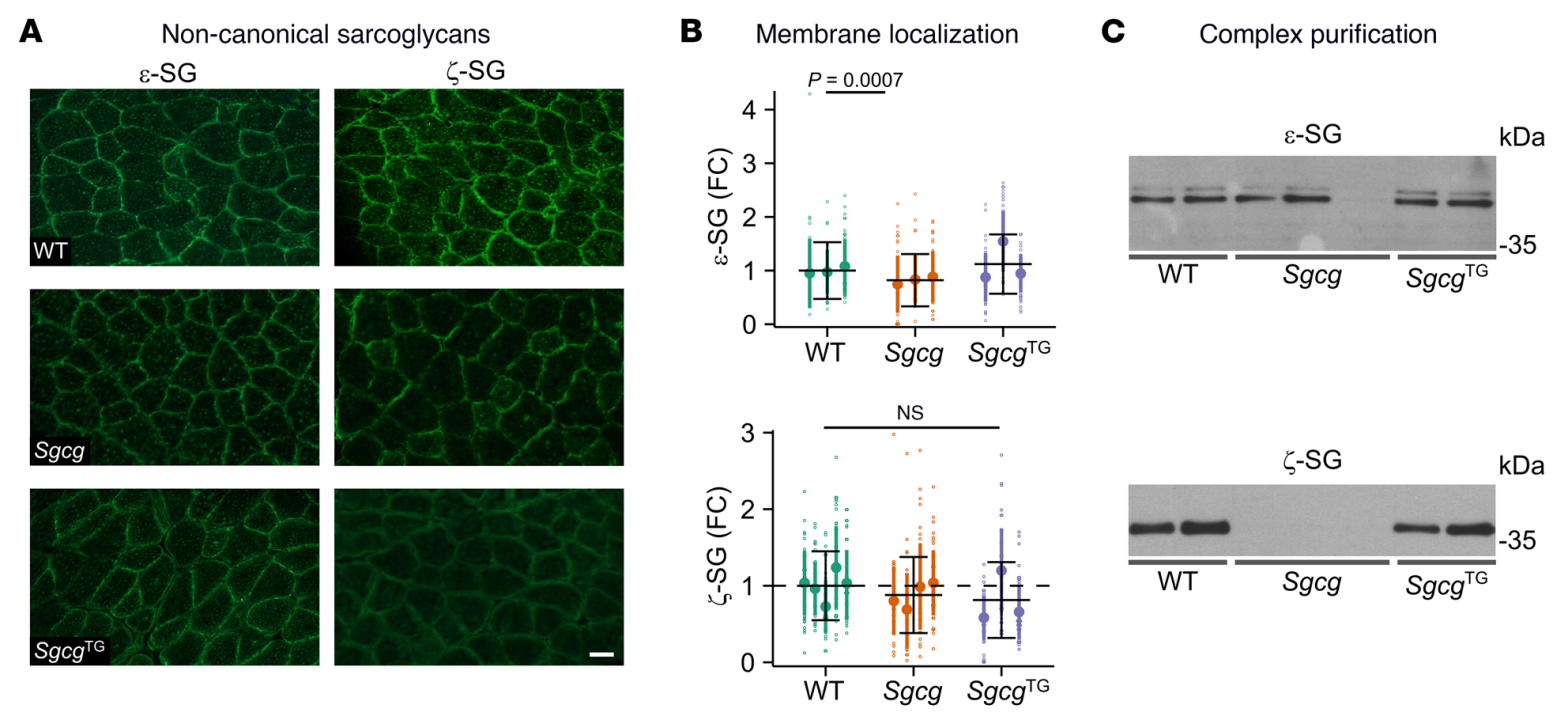
SSPN stabilizes DGC complexes containing ζ-sarcoglycan. (**A**) Indirect immunofluorescence assays of transverse WT, *Sgcg*, and *Sgcg*^TG^ quadriceps cryosections using the indicated antibodies. Scale bar: 50 μm. (**B**) Quantification of ε- and ζ-sarcoglycan abundance at the sarcolemma, *n* = 3–5 mice per genotype, 49 individual myofiber measurements per mouse (total 147–245). Data are presented as FC relative to WT (mean ± SD) for individual mice within a genotype. The mean ± SD for each genotype is provided with black solid bars (dashed line represents mean of WT samples). Statistical analysis was performed by fitting a generalized linear model. (**C**) Immunoblotting of the eluate fractions of lectin (sWGA) purifications from all 3 genotypes using the indicated antibodies (*n* = 2–3 per genotype). Proteins that interact in a complex can be detected in eluate fractions.

The prior experiments revealed that SSPN compensates for γ-SG, resulting in normal membrane localization of the SG subcomplex. Based on this, we hypothesized that SSPN induced formation of alternative SG complexes in the absence of γ-SG. We assessed ε-SG, a protein highly related to α-SG, and ζ-SG, a protein related to γ-SG. Membrane localization of ε- and ζ-SG were similar between genotypes (Figure 9, A and B). In sWGA fractions, ζ-SG was reduced in *Sgcg* but restored in *Sgcg*^TG^ muscle (Figure 9C). Reduced ζ-SG in heavy microsomes from *Sgcg* muscle, which includes the sarcolemma, has been documented previously (92). The restoration ζ-SG in *Sgcg*^TG^ sWGA lysates represents a compensatory SSPN-induced stabilization of a lower abundance SG-complex where ζ-SG takes the place of absent γ-SG, demonstrating that, in addition to sequence homology, there is functional homology between these proteins.

### SSPN restores function and integrity of the laminin-dystroglycan-dystrophin axis in γ-sarcoglycan deficiency.

**Figure 10:**
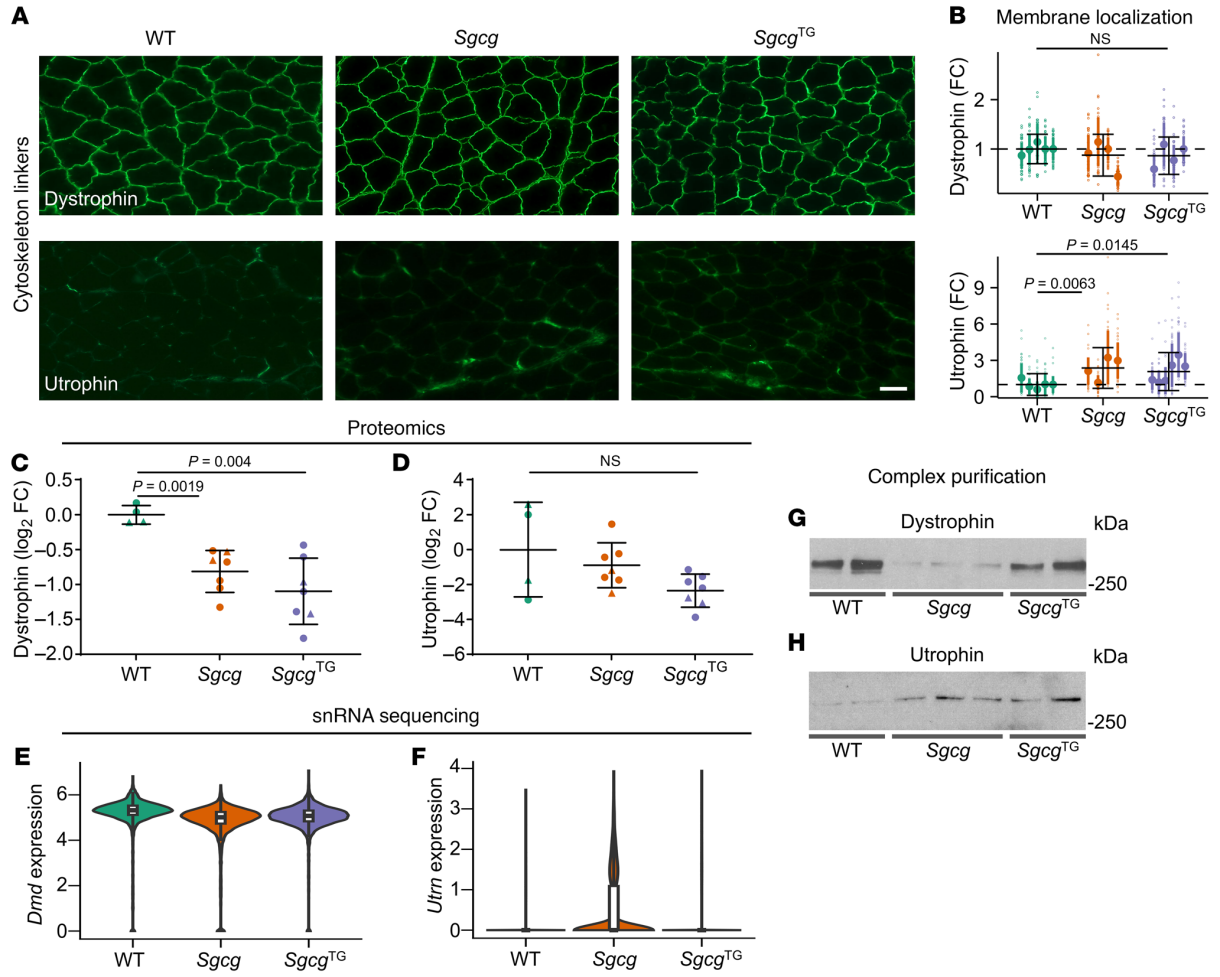
SSPN restores dystrophin-dystroglycan association. (**A**) Immunofluorescence staining and (**B**) sarcolemmal intensity quantification of dystrophin and utrophin, *n* = 4–5 mice per genotype, 49 individual myofiber measurements per mouse (total 196–245); data presented as fold change (FC) relative to WT, mean ± SD within each mouse and ± SD of all measurements per genotype. Statistical analysis by fitting a generalized linear model described in the methods section. (**C** and **D**) Relative protein levels from proteomics show dystrophin was reduced in the *Sgcg* and *Sgcg*^TG^, despite similar membrane intensity values, *n* = 4–7. Adjusted *P* values (Benjamini–Hochberg procedure) from differential protein expression analysis shown. (**E** and **F**) *Dmd* and *Utrn* mRNA expression in myonuclei from single nuclei RNA-seq. *Dmd* expression was slightly reduced in both *Sgcg* (average log_2_ FC = –0.48) and *Sgcg*^TG^ (average log_2_ FC = –0.35) compared with WT, with *Sgcg*^TG^ slightly higher than *Sgcg* (average log_2_ FC = 0.14). *Utrn* expression was elevated in *Sgcg* myonuclei (average log_2_ FC = 3.23) above WT. (**G** and **H**) sWGA enrichment of muscle lysates and immunoblotting for dystrophin and utrophin, *n* = 2–3. Dystrophin-dystroglycan association was reduced in *Sgcg* but restored in *Sgcg*^TG^ samples while utrophin-dystroglycan association was similarly increased in *Sgcg*^TG^ and *Sgcg* compared with WT.

We next assessed whether interactions with dystroglycan-associated cytoskeletal linkers, dystrophin and utrophin, were impacted in *Sgcg* and *Sgcg*^TG^ muscle. We found WT levels of dystrophin at the sarcolemma of *Sgcg* muscle (Figure 10, A and B) as previously reported (31). *Sgcg*^TG^ muscle also exhibits robust levels of sarcolemmal dystrophin (Figure 10, A and B). Sarcolemmal dystrophin in *Sgca* and *Sgcb* muscle was also similar to WT (Supplemental Figure 6A). Interestingly, sarcolemmal utrophin was increased in *Sgcg* and *Sgcg*^TG^ muscle relative to WT (Figure 10, A and B). Proteomics analysis of skeletal muscle from all 3 genotypes revealed decreased levels of dystrophin in *Sgcg* and *Sgcg*^TG^ muscle, while utrophin levels were similar (Figure 10, C and D). *Dmd* mRNA expression remained high in all genotypes, while *Utrn* expression slightly increased in *Sgcg* muscle (Figure 10, E and F), suggesting that the membrane localization of these proteins is due to improvements in protein transport or complex assembly rather than increased translation. We next assessed association of dystrophin and utrophin with dystroglycan by analysis of their abundance in sWGA eluates. We found that, while dystrophin was robustly localized at the *Sgcg* sarcolemma, it was weakly associated with dystroglycan, as revealed by its reduction in sWGA enrichments (Figure 10G). Dystrophin association with dystroglycan was restored in *Sgcg*^TG^ samples, thereby reinstating integrity of the protein-protein interactions within the DGC, including the vertical axis across the membrane, mediated by laminin-dystroglycan-dystrophin. These data also reveal that a compensatory SG-SSPN subcomplex in *Sgcg*^TG^ muscles possesses structural elements needed for dystrophin interaction either through direct interaction with dystrophin or by altering the conformation of the dystrophin binding site within the β-dystroglycan C-terminus (93) to enable dystrophin attachment. Increased association of utrophin with dystroglycan in *Sgcg* relative to weak association in WT samples (Figure 10H), together with increased membrane localization of utrophin in *Sgcg* muscles, suggests a compensatory role of utrophin in γ-sarcoglycan deficiency similar to DMD (94–96). Increased dystrophin association in *Sgcg*^TG^ coincided with increased utrophin association with the dystroglycan complex (Figure 10, G and H), suggesting that utrophin and dystrophin coexist at the *Sgcg*^TG^ skeletal muscle sarcolemma, as previously described in WT mice with moderate utrophin overexpression (97).

### SSPN binding sites are conserved in the compensatory DGC.

**Figure 11:**
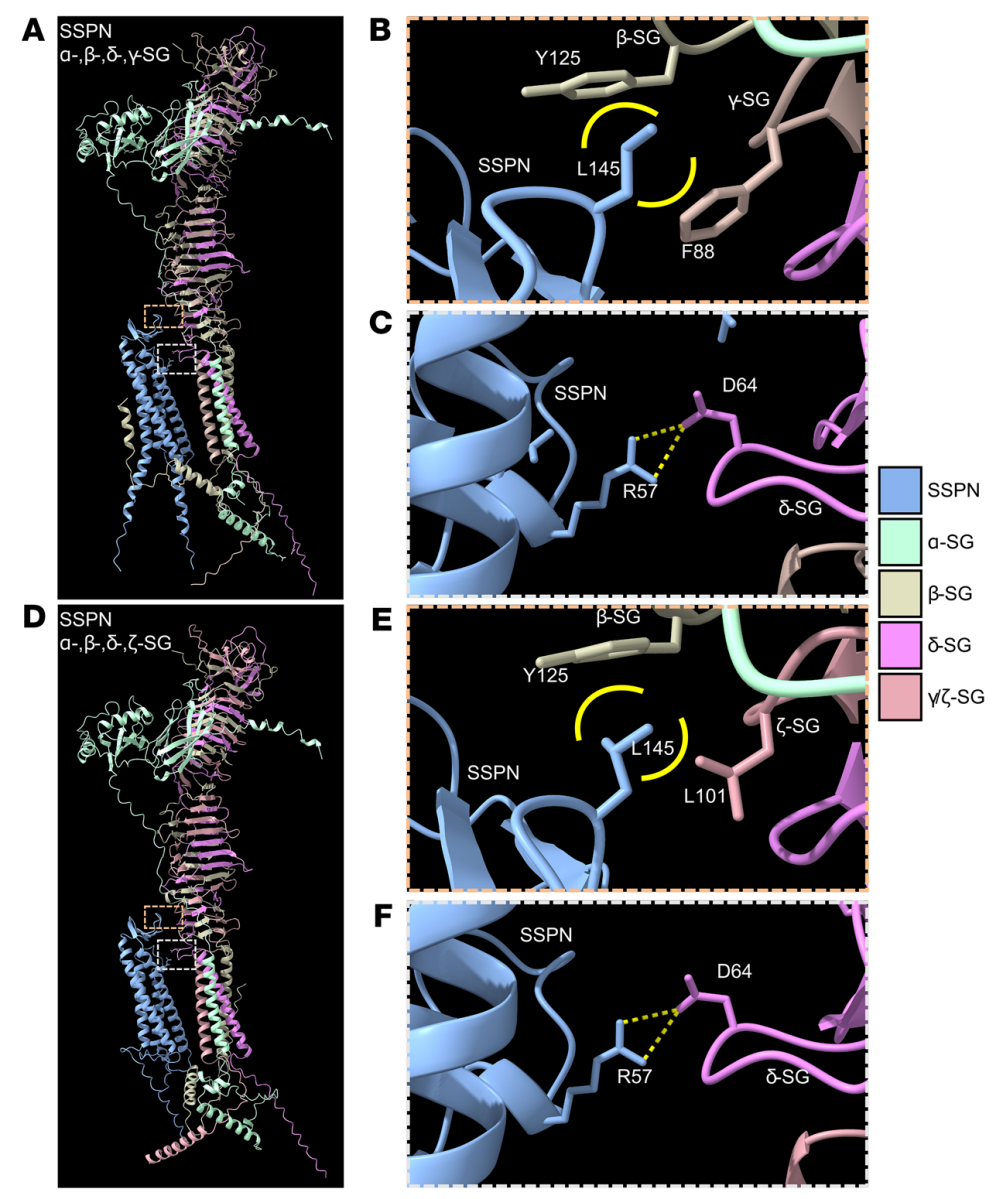
ζ-sarcoglycan replaces γ-sarcoglycan in predicted SSPN-SG structure. AlphaFold 3 predicted structures of (**A**) SSPN with α-, β-, δ-, γ-SG complex. (**B**) An inset (orange box in **A**) showing hydrophobic interactions consisting of Leu145 of SSPN in a hydrophobic pocket created by Phe88 of γ-SG and Tyr125 of β-SG. (**C**) An inset (grey box in **A**) showing a salt bridge between Arg57 of SSPN and Asp64 of δ-SG. (**D**) SSPN with α-, β-, δ-, ζ-SG complex demonstrated similarity to the canonical complex shown in **A**. (**E**) An inset (orange box in **D**) showing conserved hydrophobic interactions consisting of Leu145 of SSPN in a hydrophobic pocket created by Leu101 of ζ-SG and Tyr125 of β-SG. (**F**) An inset (grey box in **D**) showing a conserved salt bridge formed Arg57 of SSPN and Asp64 of δ-SG. Dotted yellow lines, hydrogen bonds involved in a salt bridge; yellow arcs, hydrophobic interactions.

With the finding that SSPN overexpression restores localization and formation of a compensatory SG subcomplex consisting of α-, β-, δ-, zeta-SGs, we were interested in comparing the predicted structures of the α-, β-, δ-, γ-SG and α-, β-, δ-, zeta-SG complexes and their associations with SSPN. Recent studies of sarcoglycan complex have reported an intriguing structure of the SG-SSPN subcomplex (98, 99). The extracellular portion contains a trimeric b-helix with a bend in the middle consisting of β-, δ-, γ-SG, and α-SG ascends along the β-helix with cadherin-like domain adjacent to the bend (99, 100). Since the finding of this β-helix structure, there has been no analysis of the predicted structure of SG complexes other than α-, β-, δ-, γ-SG complex, nor of their interactions with SSPN. Using AlphaFold 3 Server, we generated predicted structures of murine α-, β-, δ-, γ-SG and α-, β-, δ-, ζ-SG complexes with SSPN. The experimental structure of the DGC (98) containing SG-SSPN served as a reference for evaluating the plausibility of the predicted models. As expected, based on the 53% pairwise identity between γ-SG and ζ-SG, the general structures of the complexes were extremely similar, with ζ-SG occupying the same location as γ-SG in the trimeric bent β-helix, and α-SG maintaining its position in the structure (Figure 11, A–D). The other SGs were undisturbed between the 2 complexes, and key binding sites between the SGs and SSPN were also maintained. The large extracellular loop of SSPN has been found necessary for association between SSPN and the sarcoglycans (101), aligning with our predicted structures where Leu145 of SSPN’s large extracellular loop within the murine sequence is situated in a hydrophobic pocket on the side of the trimeric β-helix formed by Tyr125 of β-SG and Phe88 of γ-SG (Figure 11B). Leu145 is conserved in rabbit and human SSPN sequences (Supplemental Figure 9). This binding site was conserved in the α-, β-, δ-, ζ-SG structure, with the only change being Leu101 of ζ-SG replacing Phe88 of γ-SG (Figure 11E). Tyr125 of β-SG formed the other side of the hydrophobic pocket. The predicted structures also revealed another conserved binding site between the 2 complexes that has not previously been described between SSPN and the SGs. Arg57, located at SSPN’s transition from its first transmembrane loop to its small extracellular loop, was predicted to form a salt bridge with Asp64 of δ-SG at the base of the β-helix in an interaction that occurred between the same residues in both structures (Figure 11, C and F). Based on multiple sequence alignments of mammals, the participating residues of these binding sites were found to be highly conserved across species, with 97%–100% identity and 100% similarity (Supplemental Table 3). Since the function of the sarcoglycan complex is highly associated with its interactions with dystroglycan, we also compared these predicted structured in the presence of the transmembrane segment of β-dystroglycan (Supplemental Figure 10). We found a similar conservation of structure and interaction sites as in the SG-SSPN complex alone. The conserved structure and predicted interactions between the canonical and compensatory DGCs support that SSPN ameliorates pathology in *Sgcg* mice through direct binding and recruitment of ζ-SG into the SG-SSPN subcomplex, followed by assembly of dystrophin-dystroglycan at the sarcolemma.

**Figure 12:**
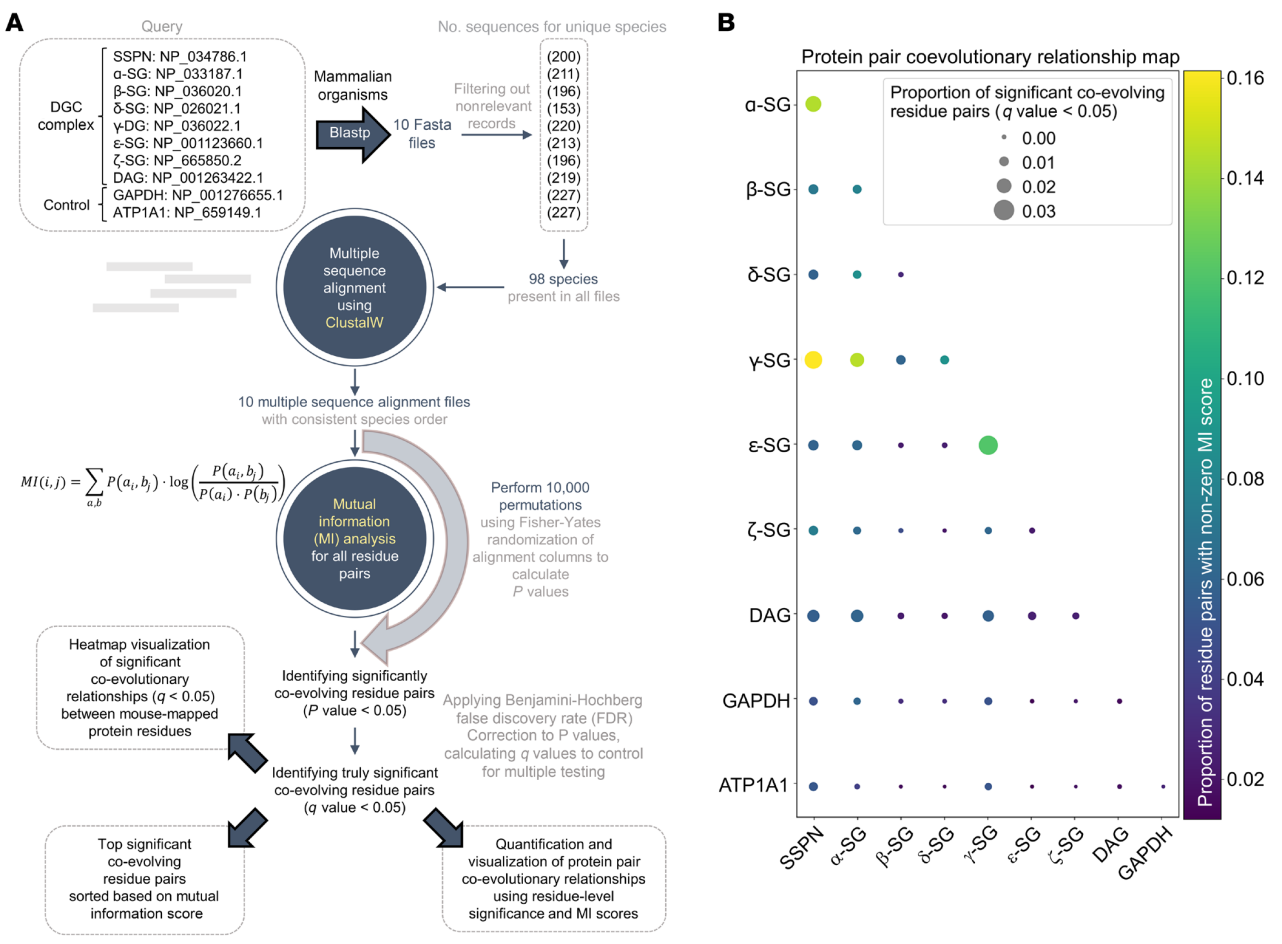
Coevolutionary analysis of protein pairs in the SG-SSPN complex and control proteins. Coevolution of residues from SSPN, α-SG, β-SG, δ-SG, γ-SG, ε-SG, ζ-SG, dystroglycan, and 2 control proteins GAPDH and ATP1A1 was analyzed. (**A**) Workflow schematic of the mutual information-based coevolutionary analysis. (**B**) Dot plot integrating mutual information scores and coevolution significance for each protein pair. Dot size represents the proportion of significantly coevolving residue pairs (*q*-value < 0.05) and color (yellow to red) indicates the fraction of residue pairs with nonzero mutual information scores. The more intense yellow color between SSPN and γ-SG indicates a higher fraction of nonzero mutual information scores for this protein pair. This analysis revealed a strong coevolutionary relationship between SSPN and γ-SG.

Based on the compensatory sarcoglycan complex underlying the *Sgcg* rescue mechanism, we next sought to determine whether phylogenic analysis could be valuable for predicting compensatory protein-protein interactions. We employed mutual information analysis using a residue-pairing approach between all components within the SG-SSPN subcomplex (Figure 12A). The most pronounced coevolutionary relationship was identified between SSPN and γ-SG, suggesting a functional association between these 2 proteins (Figure 12B). Heatmap visualization of significant coevolution relationships (*q*-value < 0.05) revealed that the highest fraction of interprotein coevolving residue pairs was between SSPN and γ-SG (Supplemental Figure 11). Furthermore, the majority of the 20 coevolving residue pairs ranked by mutual information (MI) score were between SSPN and γ-SG (Supplemental Table 4). These data suggest that the high degree of coevolution between SSPN and γ-SG may support interaction between SSPN and proteins highly similar to γ-SG, such as ζ-SG.

## Discussion

Using transgenic overexpression of SSPN, we show a robust amelioration of γ-sarcoglycan–deficient muscular dystrophy through corrected histopathology and muscle function. Proper membrane localization of the SG-SSPN subcomplex in the absence of γ-SG was associated with an upregulation of ζ-SG, which shares 74% sequence identity with γ- and δ-SG (92). The current model of the SG-SSPN complex assembly suggests that β-SG initiates the formation of the SG complex in the endoplasmic reticulum, forming a tightly associated “core” with δ-SG (58, 59). Following the β-, δ-SG “core,” α- and γ-SG are recruited for complete complex formation (58, 102). To complete the formation, SSPN likely interacts with the SG complex during transport from the Golgi apparatus in the trans-Golgi network, resulting in membrane localization of the SG-SSPN subcomplex (60).

The 6 vertebrate sarcoglycan genes evolved from 3 ancestral genes, with α- and ε-SG deriving from a prevertebrate common ancestor and δ-, γ-, and ζ-SG deriving from a different prevertebrate common ancestor (18). This is evident in invertebrates, having only 3 sarcoglycan genes corresponding to the 3 vertebrate ancestral genes. β-SG does not seem to have undergone duplication and is maintained as a single gene in both vertebrates and invertebrates. Paralogous genes generally demonstrate beneficial redundancy in protection against loss of function mutations (103). Amelioration of sarcoglycanopathies through compensation by the remaining functional sarcoglycans is likely due to these paralogous genetic relationships. This results in enough similarity between the proteins to functionally replace each other in the sarcoglycan complex. For instance, α-SG deficiency has been shown to be compensated for by replacement of α-SG with ε-SG (19). In agreement with this paralogous protective effect, we found that SSPN ameliorates *Sgcg* through recruitment of ζ-SG.

SSPN amelioration of γ-SG deficiency but not α- or β-sarcoglycan deficiency may also result from the distinct physiological roles of each SG. For instance, α-SG has been shown to harbor an ATP binding domain and ATPase activity (104, 105), and Ca^2+^ binding capacity has also been proposed (106). As mentioned above, β- and δ-SG are likely crucial in forming the SG complex. Therefore, it is conceivable that β- or δ-SG deficiency thwart the initial formation of the complex, making complete assembly impossible, in part contributing to a lack of effect with SSPN overexpression. Whether SSPN overexpression is effective in *Sgcd* mice is not known, although it would likely require ζ-SG to adopt a configuration with β-SG and γ-SG that seems unsupported by predicted sequence interactions and previous biochemical studies. Additionally, amelioration by SSPN overexpression might not occur because δ-SG is absent and does not have a known potential replacement in the complex that can form the initial core complex with β-SG (55, 107). These hypotheses are consistent with the inability of γ-SG to compensate in *Sgcd* muscle, where γ-SG is destabilized and undetectable in *Sgcd* muscle (15). Expression of SGs and the C-terminal end of dystrophin in COS-1 cells showed that dystrophin cannot be immunoprecipitated with individual SGs, but is immunoprecipitated with coexpressed β- and δ-SG, suggesting that the β- and δ-SG “core” is responsible for the interaction between the SG complex and dystrophin (108). Loss of this interaction may decrease dystrophin binding and cause DGC destabilization, providing an additional requisite role of the SG core. Biochemical and cell biological evidence is supported by patient biopsy immunofluorescence images demonstrating a complete loss of the SG complex in β- or δ-SG–deficient muscle (representing a loss of the “core” for complex formation) while α- or γ-SG deficiency can cause a reduction, but not complete ablation, of SG membrane localization (109).

**Figure 13:**
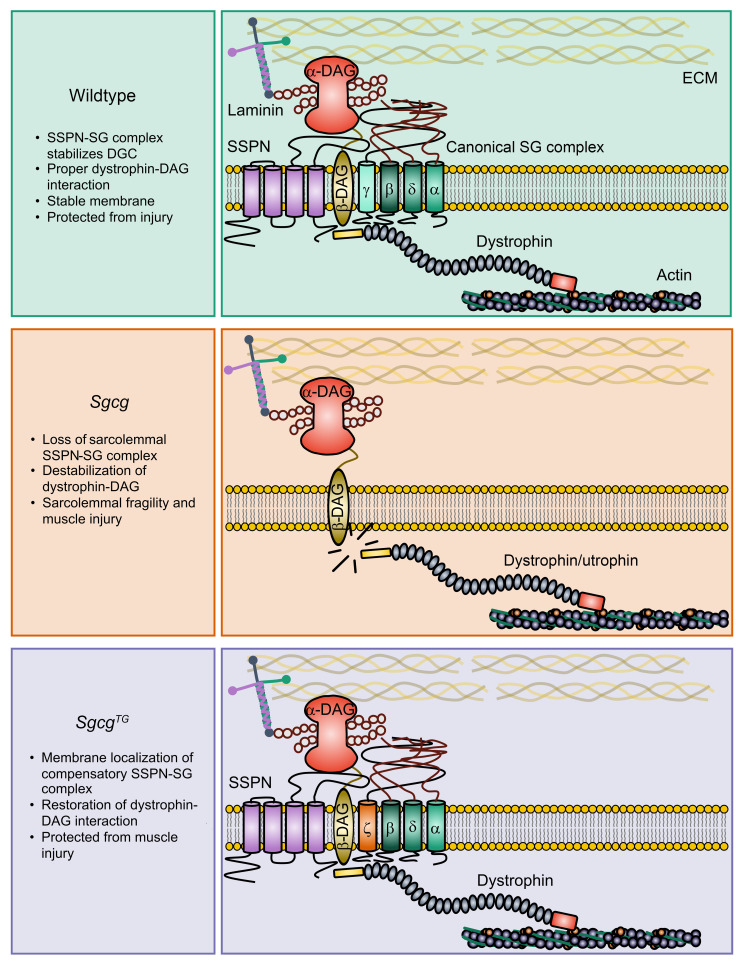
Rebuilding, rewiring and stabilization — how sarcospan prevents limb-girdle muscular dystrophy R5. The SSPN-sargoclycan complex interacts with and stabilizes the DGC and maintains dystrophin-dystroglycan association. Though expressed at lower levels in skeletal muscle, ε- and ζ-sarcoglycan form alternative sarcoglycan complexes (see discussion section for details and references). Our model suggests that SSPN overexpression restores membrane stability in γ-sarcoglycan–deficient muscle by supporting the stabilization of a lower abundance sarcoglycan complex (α-, β-, δ-, ζ-sarcoglycan). This supports restoration of dystrophin-dystroglycan binding, thereby protecting against muscle injury.

One of the notable observations in this study is the restoration of dystroglycan associated ζ-SG in *Sgcg*^TG^ muscle while it is absent or reduced in *Sgcg* mice. Diminished sarcolemmal ζ-SG in *Sgcg* and *Sgcd* mice has been reported previously (92). SSPN overexpression improves many aspects of the *Sgcg* phenotype by stabilizing a compensatory SG complex consisting of α-, β-, ζ-, δ-SG. This is supported by AlphaFold-predicted structures, showing a conservation of overall structure and of SSPN binding sites between the WT and compensatory complexes. This compensatory complex has been suggested previously by in vitro data (18). However, ζ-SG exhibits low expression in murine contractile tissue relative to γ-SG (18), likely favoring the formation of the α-, β-, γ-, δ-SG complex under physiological conditions. Our α-, β-, ζ-, δ-SG stabilization hypothesis is supported by a similar competitive nature of α- and ε-SG in the SG complex seen in C2C12 myoblasts, with the α-, β-, γ-, δ-SG being dominant (29), and the ability of ε-SG overexpression to compensate for loss of α-SG (19) while knockout of both α- and ε-SG exacerbates dystrophic severity (110). While not directly assessed in this study, it is conceivable that ζ-SG overexpression can similarly compensate for γ-SG loss by completing the mature SG complex. Microsomal α-, β-, δ-SG multimers are present in γ-SG–deficient skeletal muscle but do not localize to the membrane, suggesting a missing maturation step in the complex formation (15). This absent maturation step may be in the form of the addition of ζ-SG, stabilized by association with SSPN in *Sgcg*^TG^ muscle in this study. γ- and ζ-SG seem to both be able to associate and function as the third member of the extracellular β-helix along with the required β- and δ-SG. Given the considerable similarity between δ-, γ-, ζ-SGs, it would be interesting to investigate what parts of δ-SG make it necessary for SG complex formation in comparison to its more interchangeable paralogues. The results of this study, and prior evidence showing that ζ-SG is reduced in both γ- and δ-SG deficient microsomes (with the larger reduction occurring in δ-SG deficiency), suggests that the formation of canonical and compensatory complexes is indeed contingent upon the β-, δ-SG core, and that the low abundance α-, β-, ζ-, and δ- may be stabilized in *Sgcg*^TG^ muscle. SSPN likely provides a platform to stabilize the low abundance of the α-, β-, ζ-, δ-SG complex and its association with the DGC, localizing the complex to the membrane in the absence of γ-SG and preventing sarcolemmal fragility and muscle injury (Figure 13).

Although we found substantial improvement in muscle histology and function in *Sgcg*^TG^ mice, the smaller CSA and smaller lower muscle mass may explain the inability of *Sgcg*^TG^ mice to perform equivalent to WT mice on grip strength assessment. Nonetheless, this lack of restoration of muscle force production to WT levels does not directly indicate dystrophic muscle weakness. It may be that, while muscle strength is reduced, the lack of muscle pathology itself is indicative of otherwise healthy muscle. It is conceivable that this healthy muscle will not progressively weaken to the point of ambulatory loss and respiratory failure characteristic of dystrophic progression. The long-term effects of SSPN overexpression on disease progression will be interrogated in the future. Taken together, we show that SSPN improves hallmarks of dystrophic pathology in *Sgcg* mice and provides valuable potential rescue mechanisms, including membrane stabilization of alternative SG protein complex(es) in vivo. This study further expounds on the observation that SSPN overexpression in *mdx* mice is also effective in ameliorating disease pathology (57, 111). Prior evidence in the *mdx* model and those in *Sgcg* mice shown here implicate SSPN as a promising target in at least 2 forms of muscular dystrophy. Further development of SSPN-related therapies may enhance treatment of DMD and LGMD R5, either alone or in combination with current treatment strategies. Thus, increasing SSPN abundance, either pharmacologically or genetically, can directly target the source of dystrophic pathology by enhancing DGC and membrane stability and effectively reduce disease burden.

## Methods

### Sex as a biological variable.

Our study examined male and female mice, since the diseases modeled are relevant to both sexes. Sex was differentiated in the figures with males represented as filled circles and females as filled triangles. No formal statistical tests were conducted to parse sex differences.

All procedures are described in detail in Supplemental Material.

### Statistics.

Data are presented as mean ± SD or median, interquartile range, and minimum and maximum values (box plots). Raw data values or residuals (in the case of ANOVA) were assessed for normality using quantile-quantile plots, histograms, and Shapiro-Wilk test. For ANOVA, heteroscedasticity was assessed using Brown-Forsythe test and Bartlett’s test, and residuals were visually inspected. Unless otherwise stated in figure legends, 1-way ANOVA with Tukey’s post hoc was used for multiple comparisons. Statistical analysis of immunofluorescence intensity was conducted by fitting a generalized linear regression model using the gamma distribution family using the “identity” link function. Model fit was assessed by quantile-quantile plots (of deviance residuals) and by simulation-based diagnostics using the DHARMa package (112). To account for nonindependence/cluster-correlation resulting from multiple measurements per mouse, we employed the sandwich estimator (“sandwich” R package) (113–115), which uses clustered covariances to control for overestimation of parameter estimate precision. Pairwise comparisons with Tukey’s multiple comparisons test correction was conducted using the emmeans R package (116). Data are presented as mean ± SD within each mouse and mean ± SD of all measurements per genotype. Global proteomics differential expression analysis was performed using pairwise 2-sided *t* tests and resultant *P* values were adjusted for multiple comparisons using the Benjamini–Hochberg procedure (117). All analysis was performed with GraphPad Prism 9, R statistical software (version 4.3.2), or Python (version 3.12.5). Statistical significance was accepted for *P* less than 0.05 or *q* less than 0.05.

### Study approval.

The mice were maintained following the guidelines established by the Institutional Animal Care and Use Committee at the University of California, Los Angeles, and approval for the mice in this study was granted by the UCLA Institutional Animals Care and Use Committee (IACUC) (protocol #2000-029-61G, approval date: 08/06/2019).

### Data availability.

Data are available in supporting data files. Single nuclei RNA-seq data were deposited in the NCBI’s Gene Expression Omnibus (GEO) under accession number GSE291423. Analytic code was deposited in Github (https://github.com/dphelzer/Sgcg.git; commit ID 46a98e3). The code for mutual information analysis is available upon request from the corresponding author. Values for all data points in graphs are reported in the Supporting Data Values file.

## Author contributions

All authors worked closely as a team and contributed to the study design and methodology. EIM created sarcoglycan-deficient mice overexpressing sarcospan by cross breeding. EIM and RM performed initial histological and immunofluorescent analysis. EIM, RM, DH, JW, TSF, and JH performed histology, immunofluorescence analysis, and quantification. MM and KCH performed proteomics analysis and DH further analyzed and interpreted the proteomics data. HM designed and performed sWGA affinity purification experiments. EIM performed laminin binding assay. JW generated predicted structures of SG-SSPN complexes using AlphaFold and performed in vivo ambulation experiments and data analysis. SL and ZHZ provided the SG-SSPN complex model with dystroglycan using AlphaFold. EIM, RM, DH, HM, and JW prepared the figures, DH wrote the initial manuscript draft, and all authors reviewed and contributed revisions to the manuscript. EMM provided γ-sarcoglycan-deficient mice. MHA performed coevolution analysis with oversight from EJD. The order of the first coauthors was determined based on their contribution to the project and resulting manuscript. RHC conceptualized the project, oversaw the project, and consulted on all experiments, data analysis, and manuscript preparation. All authors reviewed and contributed revisions to the manuscript.

## Supplementary Material

Supplemental data

Unedited blot and gel images

Supporting data values

## Version 1 06/23/2025

In-Press Preview

## Version 2 09/02/2025

Electronic publication
