# Supplementary material for: Sarcospan protects against LGMD R5 via remodeling of the sarcoglycan complex composition in dystrophic mice
Source: J Clin Invest. 2025 Jun 19;135(17):e187868. doi: 10.1172/JCI187868 (PMC12404760; doi:10.1172/JCI187868)
Supplement: Supplemental data [file jci-135-187868-s269.pdf]

Sarcospan rewires the sarcoglycan complex composition to stabilize the sarcolemma and prevent limb-girdle muscular dystrophy R5

Ekaterina I. Mokhonova[1#], Daniel Helzer[1#], Ravinder Malik[1#], Hafsa Mamsa[1], Jackson Walker[1], Mark Maslanka[2], Tess S. Fleser[1], Mohammad H. Afsharinia[1], Shiheng Liu[3,4], Johan Holmberg[1,5], Z. Hong Zhou[3,4], Eric J. Deeds[1,6], Kirk C. Hansen[2], Elizabeth M. McNally[7], and Rachelle H. Crosbie[1, 8-10]*

[1]Department of Integrative Biology and Physiology, University of California Los Angeles, Los Angeles, CA, USA; [2]Department of Biochemistry and Molecular Genetics, University of Colorado Anschutz Medical Campus, Aurora, CO, USA; [3]Department of Microbiology, Immunology, and Molecular Genetics, University of California, Los Angeles, CA, USA; [4]California NanoSystems Institute, University of California, Los Angeles, CA, USA; [5]Department of Experimental Medical Science, Lund University, Lund, Sweden; [6]Institute for Quantitative and Computational Biosciences, University of California, Los Angeles, CA, USA; [7]Center for Genetic Medicine, Northwestern University Feinberg School of Medicine, Chicago, IL, USA; [8]Eli and Edythe Broad Center of Regenerative Medicine and Stem Cell Research, University of California Los Angeles, Los Angeles, CA, USA; [9]Molecular Biology Institute, University of California Los Angeles, Los Angeles, CA, USA; [10]Department of Neurology, David Geffen School of Medicine, University of California Los Angeles, Los Angeles, CA, USA

[#]Joint co-first authors

*Correspondence:
Rachelle H. Crosbie, Ph.D.
Professor and Chair
Department of Integrative Biology & Physiology
University of California Los Angeles
610 Charles E. Young Drive East
Terasaki Life Science Building
Los Angeles, CA 90095
Tel: 310-367-7905, Email: rcrosbie@physci.ucla.edu

Contents:

Supplemental Methods
Supplemental Figures 1-10
Supplemental Tables 1-4
Supplemental References

Supplemental Methods

Animal models

C57BL/6J wildtype, *Sgca*$^{+/-}$ (B6.129S6-*Sgca*^{tm2Kcam}/J), and *Sgcb*$^{+/-}$ (B6.129-*Sgcb*^{tm1Kcam}/1J) (1) mice were obtained from Jackson Laboratories (Bar Harbor, ME). *Sgcg*$^{+/-}$ mice were provided by Dr. Elizabeth McNally (Northwestern University) (2). The generation of transgenic (TG) mice harboring the full-length human SSPN (hSSPN) cDNA has been described previously (3, 4). *Sgca*^{TG}, *Sgcb*^{TG}, and *Sgcg*^{TG} mice were obtained according to the two breeding schemes outlined in Supplemental Figure 1. We crossed SG-heterozygotes (*Sgca*$^{+/-}$, *Sgcb*$^{+/-}$, *Sgcg*$^{+/-}$) to SSPN-TG mice (Supplemental Figure 1A) to generate transgenic SG-heterozygotes; then transgenic SG-heterozygotes were crossed to SG-heterozygotes to obtain transgenic SG-null mice (*Sgca*^{TG}, *Sgcb*^{TG}, and *Sgcg*^{TG}). In the second breeding scheme, we crossed SG-heterozygotes to each other to generate SG-deficient mice and then bred these mice to transgenic SG-heterozygotes (Supplemental Figure 1B). Transgenic SG-deficient mice were born at the expected ratio, 12.5% in the first breeding scheme and 25% in the second scheme. 30-week-old male mice were used for the ambulation and grip strength tests, while 12 to 20-week-old male and female mice were used for all remaining experiments. Mice were maintained in the Center for Health Sciences Vivarium and Terasaki Life Sciences Vivarium at the University of California, Los Angeles, according to approved protocols and guidelines by the UCLA Institutional Animal Care and Use Committee. Sample size was determined based on prior publications (5-8) using G*Power (9).

Co-evolutionary analysis of SG-SSPN complex proteins

Canonical protein sequences for sarcospan (NP_034786.1), a-SG (NP_033187.1), β-SG (NP_036020.1), δ-SG (NP_036021.1), γ-SG (NP_036022.1), ε-SG (NP_001123660.1), ζ-SG (NP_665840.2), dystroglycan (NP_001263422.1), GAPDH (NP_001276655.1, general control), and ATP1A1 (NP_659149.1, transmembrane control) were used as queries for BLASTP searches. To validate the specificity of the co-evolution signal, we employed a dual-control approach using GAPDH and ATP1A1. GAPDH, a widely expressed glycolytic enzyme, served as a general housekeeping control to distinguish our findings from general evolutionary patterns. ATP1A1, a ubiquitously expressed transmembrane protein essential for maintaining cellular ion gradients, provided a control for transmembrane proteins not known to be functionally related to the dystrophin-glycoprotein complex. This approach allowed us to differentiate the observed co-evolutionary relationship from both general evolutionary trends and those specific to transmembrane proteins. The NCBI Protein BLAST web server (https://blast.ncbi.nlm.nih.gov/Blast.cgi?PAGE=Proteins) was utilized with default settings, except for restricting the organism to Mammalia (taxid:40674) and setting the maximum target sequences to 5000. The resulting FASTA files were curated to retain only isoform 1 from common species across all proteins (98 records). Multiple sequence alignment was performed using ClustalW 2.1 (10). Subsequent analyses were performed using Python 3.12.5.

Mutual information (MI) was calculated for all intra-protein and inter-protein column pairs in the alignment files using the formula:

$$MI(X,Y) = \sum p(x,y) \times \log_2\left(\frac{p(x,y)}{p(x) \times p(y)}\right)$$

High MI scores between columns suggest co-evolution, potentially indicating functional relationships between those positions in the proteins (11). MI scores were mapped to mouse protein residue pairs, excluding columns with gaps in mouse proteins. To ensure the observed MI scores were significantly higher than expected by chance, 10,000 permutation tests were

performed using Fisher-Yates randomization. P-values were calculated for each residue pair. The Benjamini-Hochberg procedure was applied to control the false discovery rate. Significant co-evolving residue pairs were identified using a q-value threshold of 0.05.

To ensure that the observed co-evolution was not constrained by individual residue conservation, the information content (IC) was calculated for each residue using the following formula:

$$\mathrm{IC} = \log_2(20) + \sum p(x) \log_2(p(x)),$$

where $p(x)$ is the frequency of amino acid x at a given position, and $\log_2(20)$ represents the maximum possible entropy for protein sequences (as there are 20 standard amino acids). The IC score ranges from 0 to 4.2 bits, with higher values indicating greater conservation. Moderate IC scores allow for sufficient variability to be detected by mutual information analysis without being constrained by individual conservation.

Significant co-evolving residue pairs were visualized using a heatmap. The proportion of significant co-evolving residue pairs and the proportion of residue pairs with non-zero MI scores were calculated for each protein pair and visualized using a dot plot. The top significant co-evolving residue pairs were identified based on MI scores and IC values were also calculated, ensuring that the observed co-evolution was not simply a result of high individual residue conservation.

Indirect immunofluorescence staining

Freshly dissected muscles were mounted in OCT (Tissue-Tek), frozen in liquid-nitrogen chilled isopentane, and stored at -80°C for future analysis. Transverse 10 μm quadriceps cryosections were blocked in 3% BSA in PBS for 1 hour at room temperature. The avidin/biotin blocking kit (SP-2001; Vector Laboratories) and the Mouse on Mouse (M.O.M.) immunodetection kit (BMK-2202; Vector Laboratories) were used according to the manufacturer's instructions for primary antibodies raised in mouse. Sections were incubated with the following primary antibodies in 3% BSA in PBS or M.O.M. Diluent at 4°C overnight: dystrophin (MANDYS8; D8168, 1:200; Sigma-Aldrich), utrophinM3 (MANCHO3 84A, 1:5; Developmental Studies Hybridoma Bank), a-sarcoglycan (NCL-L-a-SARC, 1:100; Leica Biosystems), β-sarcoglycan (NCL-L-b-SARC, 1:20; Leica Biosystems), γ-sarcoglycan (GTX117176, 1:4000; GeneTex), δ-sarcoglycan (ab137101, 1:100; Abcam), ε-sarcoglycan (2407-1-AP, 1:50; Thermo Fisher Scientific), ζ-sarcoglycan (NBP2-48536, 1:50; Novus Biologicals), β-dystroglycan (NCL-L-b-SARC, 1:20; Leica Biosystems), α-dystroglycan (DGIIH6 C4s, 1:20; Developmental Studies Hybridoma Bank), β1D integrin (MAB1900, 1:30; Millipore), laminin (L9393; 1:200; Sigma-Aldrich), spectrin (PA5-52970, 1:200; Thermo Fisher Scientific), eMHC (F1.652, 1:25; Developmental Studies Hybridoma Bank), SSPN E2 (sc-393187; 1:50; Santa Cruz Biotechnology), type I collagen (CL50151AP-1; 1:250; Cedarlane Labs), and laminin alpha-2 (AB11576; 1:150; Abcam). Primary antibodies were detected by biotinylated anti-mouse IgG (BA-9200; 1:500; Vector Laboratories) or biotinylated anti-rabbit IgG (BA-1000; 1:500; Vector Laboratories) and fluorescein–conjugated avidin D (A-2001; 1:500; Vector Laboratories). The M.O.M. block was excluded for IgG membrane permeability staining. Laminins (L9393 and AB11576) were detected with goat anti-rabbit Alexa Fluor 594 (A-11037, 1:500; Invitrogen). Sections were mounted with Vectashield with or without DAPI (Vector Laboratories). Images were captured with a Zeiss Axio Imager M2 (Carl Zeiss Inc., Thornwood, NY, USA) with a Hamamatsu ORCA-Flash 4.0 V3 digital complementary metal oxide semiconductor camera and a plan-Apochromat 20×/0.8 M27 objective. Exposure times were kept the same between compared

genotypes. Image analysis was performed using ImageJ 1.50i software (NIH), QuPath (12), or Zen (Carl Zeiss).

Histology

Central nucleation, muscle fiber cross-sectional area, and diaphragm thickness were visualized by H&E staining of 10 μm thick transverse cryosections as described previously (13). The percentage of fibers with central nucleation was calculated using 3-4 fields of view per cross-section, and for every region of view ~500 fibers were manually counted, totaling 1500-2000 fibers per muscle section. Diaphragm thickness was measured in ImageJ by taking an average of five measurements equally spaced along the length of the H&E-stained muscle section. Myofiber CSA was measured from 500 random, clearly demarcated fibers per sample (14) from whole quadriceps sections.

Picrosirius red staining for collagen deposition

Transverse quadriceps and diaphragm cryosections (10 μm) were stained with Picro-Sirius Red using a commercial kit (KTPSRPT; StatLab), according to the manufacturer's protocol with the following modifications developed for cryo-sections. Slides were acclimated at room temperature (RT) for 10 min and rehydrated through a series of graded alcohol washes (100%, 80% and 50%), 2 min in each solution. Sections then were soaked in distilled water for 2 min and fixed with 4% PFA/PBS for 30 min at RT, followed by two washing in distilled water, 5 min each. After staining with Wiegert hematoxylin for 5 min, slides were washed for 3 minutes in three changes of distilled water and stained in Picro-Sirius Red for 1 hour. Slides were then washed twice in 0.5% acetic acid, 1 min each wash, submersed in 90% ethanol for 30 sec, quickly dehydrated in 100% ethyl alcohol (2 changes, 30 sec each), cleared with xylene (2 changes, 5 min each), and mounted with Permount mounting medium (UN1294; Thermo Fisher Scientific). Images were captured using the same microscope setup as noted previously for indirect fluorescence analysis, except that they were widefield. The fibrotic area was measured using ImageJ 1.50i software, with each muscle section having its largest contiguous region manually drawn as a ROI and the total area in pixels of the ROI was measured. This ROI was drawn along the outside edge of an area one myofiber internal to the epimysium so that only collagen internal to the muscle was measured. The Color deconvolution 2 (15) plug-in for ImageJ was used on an RGB image to unmix the colors, with the pale-yellow color of the cytoplasm being subtracted and the red staining of collagen being kept. The output image was converted to 16-bit and the threshold function was used to exclude any remaining intracellular regions and generate a binary image representing pixels positive for collagen staining. The same manually drawn ROI was used on top of this binary image to measure the amount of collagen positive pixels, which was divided from the total pixels of the ROI to get collagen percent area.

IgG^+ permeabilized and $eMHC^+$ newly regenerated fibers

Number of IgG or eMHC positive fibers was determined by counting positively stained fibers demarcated with laminin and dividing by the quadriceps image cross-sectional area (8).

Membrane protein intensity analysis

Immunofluorescence images were quantified using the line drawing tool on ImageJ 1.50i software. Lines were drawn perpendicular to the cell membrane, and peak intensity values were plotted, denoted as "max" measurements on ImageJ. For random sampling of the images, ImageJ's

grid tool with random offset was used with the myofiber membrane closest to each grid crossing being measured. The background signal (averaged signal from the unstained centers of 5 random muscle fibers) was subtracted from each max measurement of the stained areas to get the corrected max value used for analysis. Forty-nine non-overlapping cross-sectional areas per animal were quantified.

Creatine kinase assay

Plasma was collected during tissue harvest by cardiac puncture. Immediately after collection samples were snap-frozen in liquid nitrogen and kept at -80°C. Before analysis, samples thawed on ice and diluted 1:100 in 0.9% NaCl. The creatine kinase assay and data analysis were performed by BioAssay Systems using EnzyChrom™ Creatine Kinase Assay Kit (ECPK-100). In a clear bottom 96-well plate 10 µL of each sample and 100 µL of reagent per well was loaded in triplicates. Plates were incubated for 20 min at room temperature and read OD_{340nm} at 20 min and again at 40 min. CK activity was calculated using the following equation:

$$\text{CK (U/L)} = \frac{OD_{40min} - OD_{20min}}{OD_{Calibrator} - OD_{H_2O}} \times 150 \times n,$$

where n is the dilution factor. OD_{40min} and OD_{20min} are OD_{340nm} values at 40 min and 20 min for the sample. $OD_{Calibrator}$ and OD_{H2O} are OD_{340nm} values of the Calibrator and water blank at 40 min. The value 150 is the equivalent activity (U/L) of the Calibrator under the assay conditions.

sWGA enrichment

Skeletal hindlimb muscles (excluding quadriceps, gastrocnemius, and tibialis anterior muscles) were snap frozen and stored at -80°C until analysis. Tissues were pulverized to a fine powder using a chilled BioPulverizor (59014N; BioSpec). Protein was extracted by Dounce homogenizing in 10x volume of chilled digitonin lysis buffer (50 mM Tris, 500 mM NaCl, 1% digitonin, pH 7.8) containing Halt protease (1860932; Thermo Scientific) and Halt phosphatase inhibitors (78428; Thermo Scientific) and solubilized for 1 hour at 4°C. Lysates were clarified by centrifugation at 20,000 x*g* for 20 min at 4°C. The DC Protein Assay (5000113-115; Bio-Rad) was used to determine protein concentration. Succinylated Wheat Germ agglutinin conjugated agarose beads (sWGA; AL-1023S-5; Vector Laboratories) pre-equilibrated with digitonin lysis buffer were rotated with equal amounts of protein (10 mg) overnight at 4°C. The following day, beads were spun at 1,000 x*g* for 5 min and rinsed five times with digitonin wash buffer (50 mM Tris, 500 mM NaCl, 0.1% digitonin, 0.2 mM PMSF, 0.75 mM benzamidine, 5 mM calpain I and 5 mM Calpain II, pH 7.8). Bound proteins were eluted with digitonin wash buffer containing 0.3 M GlcNAc (N-acetyl glucosamine; A8625; Sigma-Aldrich) for 1 hour at 4°C before concentrating with the 10 kDa filtration columns Amicon Ultra-15 (UFC901024; Millipore). sWGA eluates were stored at -80°C prior to analysis. All incubation and centrifugation steps were performed at 4°C.

Immunoblot analysis

Equimolar amounts of sWGA eluates (10-100 µg) were separated on 4-12% gradient Bolt Bis-Tris gels (NW04122BOX; Thermo Scientific) and transferred onto nitrocellulose membranes (926-31092; Li-Cor Biosciences). PageRuler Plus Prestained Protein Ladder (PI26619; Thermo Scientific) was used as a molecular weight marker. Membranes were blocked with 5% non-fat dry milk (Carnation) in TBST (50 mM Tris, 100 mM NaCl, 0.1 % Tween-20, pH 7.5) for 1 hour at RT followed by primary antibody incubation overnight at 4°C with gentle rotation. The following primary antibodies were diluted in blocking buffer: dystrophin (MANDYS8; D8168, 1:500; Sigma-Aldrich), α-sarcoglycan (NCL-L-a-SARC, 1:100; Leica Biosystems), β-sarcoglycan

(NCL-L-b-SARC, 1:75; Leica Biosystems), γ-sarcoglycan (GTX117176, 1:4000; GeneTex), δ-sarcoglycan (ab137101, 1:600; Abcam), ε-sarcoglycan (2407-1-AP, 1:100; Thermo Fisher), ζ-sarcoglycan (NBP2-94447, 1:400; Novus Biologicals), β-dystroglycan (MANDAG2 7D11, 1:500; Developmental Studies Hybridoma Bank), α-dystroglycan (DGIIH6 C4s, 1:50; Developmental Studies Hybridoma Bank), human SSPN (LS-C747357, 1:30,000; LifeSpan Bioscience), and mouse SSPN (Crosbie Lab 10B8, 1:2,000). Immunoblots were extensively rinsed in TBST before applying horseradish peroxidase-conjugated anti-mouse IgG (ab6789, 1:40,000; Abcam), anti-rabbit IgG (ab6721, 1:40,000; Abcam) or anti-mouse IgM (626820, 1:10,000; Thermo Scientific) secondary antibody in blocking buffer for 1 hour at room temperature. Bound antibodies were detected with SuperSignal West Pico Plus Chemiluminescence (34580; Thermo Scientific) and signal developed on GeneMate Blue Autoradiography Film (F-9023; GeneMate).

Solid phase binding assay

For laminin binding solid phase assay, 96-well Nunc MaxiSorp plates (Invitrogen) were coated with 100 μL of sWGA eluates overnight at 4°C in bicarbonate buffer. All subsequent steps were performed at room temperature. After three washes with 0.5% BSA in Laminin Binding Buffer (LBB) (10 mM triethanolamine, 140 mM NaCl, 1 mM $CaCl_2$, 1 mM $MgCl_2$, pH 7.6), wells were blocked with 3% BSA in LBB for two hours, washed again before incubation with varying concentration of laminin (0.15 – 90nM) in 3% BSA in LBB for another two hours. After washing unbound laminin with 0.5% BSA in LBB (three times, 5 min each), plates were incubated with anti-laminin antibodies (L9393; Sigma Aldrich) for one hour and washed with 0.5% BSA in LBB as described above. HRP-conjugated goat anti-Rabbit IgG (Abcam) was added to the wells and incubated for another hour. Unbound antibodies were washed away with 0.5% BSA in LBB and signal developed using tetramethylbenzidine (1-Step Ultra TMB-ELISA Substrate Solution, Thermo Scientific). The reaction was stopped with 2 M sulfuric acid and absorbance read at 450 nM using a Spectramax M2 microplate reader (Molecular Devices).

Grip strength

The forelimb grip strength was measured using a digital force gauge (DFS II, Ametek, Berwyn, PA) connected to a horizontal pull bar. Ten trials were performed consecutively for each mouse, with a 30-second rest period between each trial. Each trial consisted of lifting the mouse by the tail so that it grasped the pull bar and then gently pulling the mouse away until it let go of the bar. This was repeated five times for each trial, then the peak force in Newtons was recorded. The peak force was then divided by the mouse's weight in grams to normalize for body mass. These and the open-field activity methods are minor modifications of those previously described (16).

Open field activity

Ambulation assays were performed as described previously (16). 30-weeks old wildtype, *Sgcg*, and *Sgcg*^{TG} males were first challenged with an exercise protocol consisting of 10 consecutive grip strength trials. Immediately after the final forelimb grip strength trial, each mouse was placed in an individual chamber and recorded for 6 min using a smartphone camera. Each video was converted into 15 frames per second MP4 file using VLC media player (Version 3.0.19, https://www.videolan.org/vlc). Then, the distance the mouse moved was tracked frame by frame using the open-source quantitative video analysis tool Kinovea (Version 0.9.5, https://www.kinovea.org/).

Sample preparation for proteomic analysis

Quadriceps muscles were collected from 8-20 weeks old mice and snap-frozen in liquid nitrogen. Tissues snap-frozen in liquid nitrogen were milled with a mortar and pestle prior to lyophilization. All following extraction buffers were applied at 200 μL/mg of the starting tissue dry weight. Five milligrams of lyophilized material from each sample was homogenized in decellularization buffer (50 mM Tris-HCl (pH 7.4), .25% CHAPS, 25mM EDTA, 3M NaCl) at power 8 for 3 min (Bullet Blender, Model BBX24, Next Advance, Inc.) before being vortexed for 20 min at 4°C (power 7). Homogenate was spun at 18,000 x g (4°C) for 15 min and the supernatant was collected. Decellularization was repeated for a total of 3 washes, homogenizing and vortexing samples before each collection, and all washes were pooled to generate the cellular fraction. The remaining pellets were homogenized in 6 M guanidine hydrochloride (Gnd-HCl), 100 mM ammonium bicarbonate (ABC) at power 8 for 1 min and vortexed (power 5) at room temperature overnight. Homogenate was spun at 18,000 x*g* (4°C) for 15 min and the supernatant was collected as the soluble ECM (sECM) fraction. Pellets were then treated with freshly prepared hydroxylamine (HA) buffer (1 M NH_2OH−HCl, 4.5 M Gnd−HCl, 0.2 M K_2CO_3, pH adjusted to 9.0 with NaOH). Samples were homogenized at power 8 for 1 min and incubated at 45°C with shaking (1000 rpm) for 4 h. Following incubation, the samples were spun for 15 min at 18,000 x *g* and the supernatant was removed and stored as the insoluble ECM (iECM) fraction. All collected fractions were immediately frozen and stored at −80°C until further proteolytic digestion. For quantitative analysis, approximately 30 μg of protein from each fraction was combined with 500 fmol of stable isotope labeled quantitative concatemers (QconCATs (17, 18)) representing ECM, ECM-associated, and cellular proteins of interest (19) prior to digestion. Fractions were subsequently subjected to enzymatic digestion overnight (16 h) at 37°C with trypsin (1:100 enzyme to protein ratio) using a filter aided sample preparation (FASP) approach as previously described (20). For quantitative analysis, final volume was adjusted to inject 6 μg of protein and 100 fmol of QconCAT standard for each run.

LC-MS/MS analysis for global proteomics

Digested peptides were loaded onto individual Evotips following the manufacturers protocol and separated on an Evosep One chromatography system (Evosep, Odense, Denmark) using a Pepsep column, (150 μm inner diameter, 15 cm) packed with ReproSil C18 1.9 μm, 120 Å resin. Samples were analyzed using the instrument default "30 samples per day" LC gradient. The system was coupled to the timsTOF Pro mass spectrometer (Bruker Daltonics, Bremen, Germany) via the nano-electrospray ion source (Captive Spray, Bruker Daltonics). The mass spectrometer was operated in PASEF mode. The ramp time was set to 100 ms and 10 PASEF MS/MS scans per topN acquisition cycle were acquired. MS and MS/MS spectra were recorded from m/z 100 to 1700. The ion mobility was scanned from 0.7 to 1.50 Vs/cm^2. Precursors for data-dependent acquisition were isolated within ± 1 Thomson and fragmented with an ion mobility-dependent collision energy, which was linearly increased from 20 to 59 eV in positive mode. Low-abundance precursor ions with an intensity above a threshold of 500 counts but below a target value of 20000 counts were repeatedly scheduled and otherwise dynamically excluded for 0.4 min.

Global proteomics data analysis

Data was searched using MSFragger via FragPipe v20.0. Precursor tolerance was set to ±15 ppm and fragment tolerance was set to ±0.08 Da. Data was searched against SwissProt restricted to *Mus musculus* with added common contaminant sequences (17,314 total sequences). Enzyme

cleavage was set to semi-specific trypsin for all samples. Fixed modifications were set as carbamidomethyl (C). Variable modifications were set as oxidation (M), oxidation (P) (hydroxyproline), deamidation (NQ), Gln->pyro-Glu (N-term Q), and acetyl (Peptide N-term). Label free quantification was performed using IonQuant v1.10.12 with match-between-runs enabled and default parameters. Cellular, sECM, and iECM fractions were searched separately and merged after database searching. Results were filtered to 1% FDR at the peptide and protein level. Further analysis was conducted using R Statistical Software to process raw intensity values. Using the DEP package (21), data was filtered by removing proteins present in less than 2/3 of the samples. The remaining raw intensity values were normalized by variance stabilizing normalization (vsn) (22). Missing values were graphically visualized using the standard DEP guide and treated as missing not at random (MNAR) due to seemingly systemic missing values in wildtype samples and the observation that missing values appeared biased towards lower intensities, suggesting these proteins are near the detection limit. Missing values were imputed using the "MinProb" method in DEP, borrowed from the MSnbase package (23) under the assumption that the majority of missing values are MNAR, of which left-centered imputation methods exhibit superior performance. The mean normalized intensity of wildtype samples was subtracted from each value to represent the data as $\log_2$ fold change since vsn approximately corresponds to a $\log_2$ transformation, with the benefits of no singularity at zero and "calibrated" intensities to promote variance stabilization (22, 24).

LC-MS/MS analysis for quantitative proteomics analysis

Quantitative analysis was carried out by liquid chromatography – selected reaction monitoring (LC-SRM) analysis on a QTRAP®5500 triple quadrupole mass spectrometer (ABSciex) couple with a UHPLC Ultimate 3000 (Thermo Fisher) as previously described (25). Each sample was injected and separated by reversed phase chromatography (Waters, Acquity UPLC BEH C18, 1.7 µm 150 × 1 mm) by running a gradient from 2% to 28% acetonitrile in 0.1% formic acid for 28 min at a flow rate of 150 µL/min. The mass spectrometer was run in positive ion mode with the following settings: source temperature of 210°C, spray voltage set to 5300 V, curtain gas of 20 psi, and source gas of 35 psi (nitrogen gas). Data were acquired using the instrument-controlled software, Analyst (v1.6.2). QconCAT transition selection, declustering potential, collision energies, and retention times were specifically optimized for each peptide of interest using the Skyline software package (26) and settings can be found in previously published data (27).

Quantitative proteomics data analysis

For targeted LC-SRM runs, files were directly loaded into the Skyline software package (version 4.2) (26). Peaks were manually validated and light to heavy ratios (12C6/13C6) for each target peptide were collected as previously described (27).

Single nuclei isolation

Quadriceps muscle from 8-12 weeks old female mice were frozen in liquid nitrogen and stored in liquid nitrogen until use. One quadriceps from wildtype, *Sgcg*, and *Sgcg*^{TG} mice were processed for sequencing. Frozen quadriceps were briefly incubated on ice then submerged in lysis buffer (10 mM Tris-HCl pH 7.4 (T2194; Sigma), 10 mM NaCl (59222C; Sigma), 3 mM $MgCl_2$ (M1028; Sigma), 0.1% NP40 (I8896, Sigma), 1 mM dithiothreitol (646563; Sigma), 1 U/µL Roche RNase inhibitor (3335402001, Sigma)), minced with scissors, then dounced in lysis buffer. Samples were centrifuged at 500 x*g* for 5 min at 4°C and washed in BSA solution (1% BSA (130-091-376,

Miltenyi Biotec) in PBS with 1 U/µL RNase inhibitor). Samples were then centrifuged at 100 x*g* for 1 min at 4°C to pellet large debris. The supernatant was then filtered through 70 µm and 30 µm filters and centrifuged at 500 x*g* for 5 min at 4°C. Pellets were resuspended in BSA solution and nuclei were stained with 7-Aminoactinomycin D (7-AAD; 1:100 from 1 mg/mL stock; SML1633, Sigma) for 5 min on ice. Pellets were centrifuged and washed in BSA solution. 7-AAD$^+$ nuclei were isolated by FACS (UCLA BSCRC flow cytometry core). Sorted nuclei were then permeabilized in permeabilization buffer (10 mM Tris-HCl pH 7.4, 10 mM NaCl, 3 mM $MgCl_2$, 0.01% Tween-20 (1662404, BioRad), 1% BSA, 0.01% NP40, 0.001% digitonin (BN2006, Thermo Fisher Scientific), 1 mM dithiothreitol, 1 U/µL RNase inhibitor) for 2 min on ice. Nuclei were diluted in wash buffer (10 mM Tris-HCl pH 7.4, 10 mM NaCl, 3 mM $MgCl_2$, 0.1% Tween-20, 1% BSA, 1 mM dithiothreitol, 1 U/µL RNAase inhibitor) and centrifuged at 500 x*g* for 5 min at 4°C. Nuclei were resuspended in 1× nuclei buffer (20× nuclei buffer (PN-2000153, 10X Genomics), 1 mM dithiothreitol, 1 U/µL RNase inhibitor), counted, and adjusted to 3000-8000 nuclei/µL.

Single nuclei library construction, sequencing, and analysis

Nuclei suspensions were submitted to the Technology Center for Genomics and Bioinformatics core at UCLA and sequenced using the 10X Genomics platform. The single cell library was prepared using Chromium Single cell 3' Library Construction. Samples were then sequenced using Illumina NovaSeq S1 (2×50, 1 lane). The data was first processed using the 10X Genomics CellRanger pipeline. The resultant filtered count matrices were analyzed using Seurat (28) in R statistical software. Quality control steps and annotations were conducted on each sample individually using the Seurat pipeline with minor modifications. An initial filtering step was performed to remove the top and bottom 2% of nuclei based on total UMI counts. Dimensionality reduction was conducted using variable genes identified through the SCTransform() Seurat function. Clustering was performed using the FindClusters() function in Seurat using the Leiden algorithm (29). Clusters were annotated using gene markers identified in previously published literature (30). Gene expression counts were normalized by dividing each gene count by the total UMI counts for that cell, multiplying this value by 10,000, adding 1, and taking the natural logarithm. Of the cell types identified, only myonuclei were selected for gene expression visualization in this study. Normalized gene counts in myonuclei are presented in the figures.

***In silico* structural analysis**

The full-length amino acid sequences and post-translational modifications of sarcospan (UniProt entry: Q14714), b-SG (P82349), d-SG (P82347), γ-SG (P82348), ζ-SG (Q8BX51), and a-SG (P82350) excluding signal peptide from *Mus musculus* were retrieved from UniProt.org and used for modeling with the AlphaFold 3 Server. The b-DAG containing structure (Supplemental Figure 10) did not contain protein post-translational modifications. For the α-, β-, δ-, γ-SG complex, sequences for all proteins except for ζ-SG were submitted as a job, while for the α-, β-, δ-, ζ-SG complex, sequences for all proteins except for γ-SG were submitted as a job. For each complex, the CIF file corresponding to the model with the highest-ranking confidence score was chosen. The CIF files were opened in UCSF ChimeraX, where all structural graphics and analyses were performed in reference to the experimental structure of SG-SSPN to ensure model validity (31). For each structure, hydrophobic interactions were found using the contacts tool in ChimeraX to find contacts between sarcospan and the sarcoglycans between two hydrophobic residues with buried solvent-accessible surface area ≥ 15 Å^2. For salt bridges, the contacts tool was used to find oppositely charged residues between sarcospan and the sarcoglycans with atomic distances

between them being ≤ 3 Å. Hydrogen bonding between such residues was then set to show only hydrogen bonding involved in salt bridges with a 0 Å distance tolerance and 20° angle tolerance.

The structures of β-DG (transmembrane and juxtamembrane fragments: aa. 736-773, UniProt Q62165) in complex with SSPN-SG were also predicted using AlphaFold 3. To minimize interference from unstructured regions, the cytoplasmic fragments of α-, β-, γ-, and δ/ζ-SG were excluded during prediction. The structural analysis of the β-DG-SSPN-SG complexes follows the same approach as that of the SSPN-SG complexes described above.

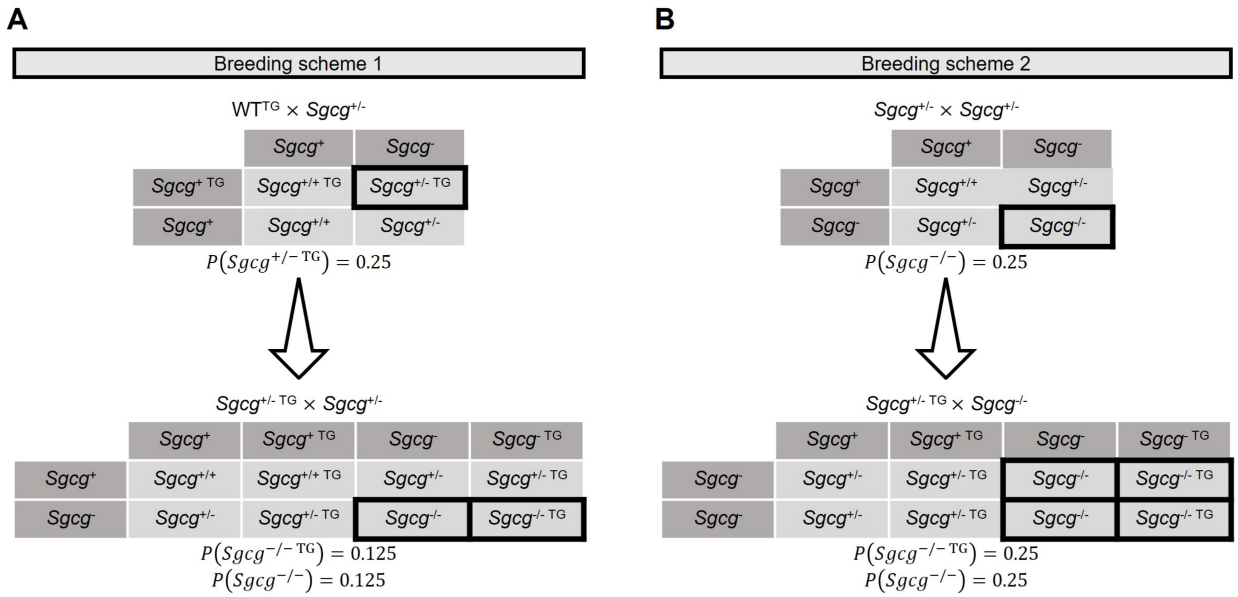

Supplemental Figure 1. Generation of sarcoglycan-deficient mice overexpressing SSPN. Two breeding schemes depicting the genotypes of male and female breeders as well as their offspring are shown. Both approaches were used in this study. While *Sgcg* mice are shown as an example in the illustration, an identical approach was used to generate *Sgca*^{TG} and *Sgcb*^{TG} mice. The probability of generating sarcoglycan-deficient mice overexpressing SSPN are provided. *Sgcg*$^{-/-}$ and *Sgcg*$^{-/-\ \mathrm{TG}}$ mice are referred to as *Sgcg* and *Sgcg*^{TG} in the manuscript text.

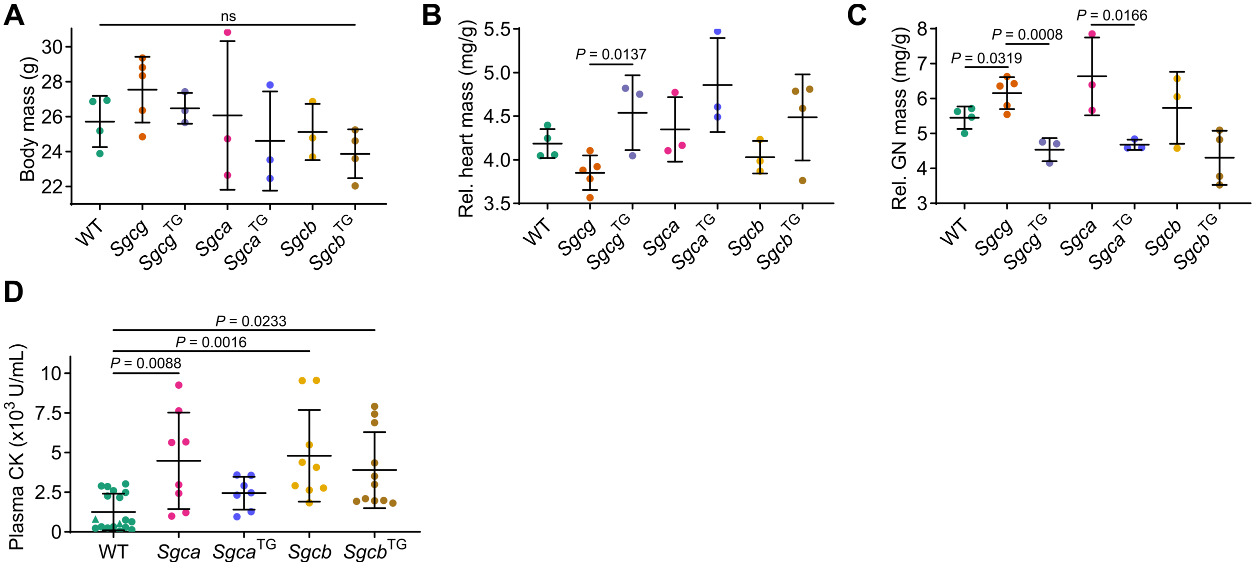

Supplemental Figure 2. Analysis of body mass, striated muscle mass, and creatine kinase release in *Sgca* and *Sgcb* mice overexpressing SSPN. (**A**) Body mass, (**B**) relative heart mass, and (**C**) relative gastrocnemius (GN) mass for the indicated genotypes (wildtype, *Sgcg*, *Sgcg*^{TG}, *Sgca*, *Sgca*^{TG}, *Sgcb*, and *Sgcb*^{TG}). Overexpression of SSPN decreased body and muscle mass across all three genotypes. Significant reduction of relative muscle mass was observed in *Sgca*^{TG} and *Sgcg*^{TG} mice compared to non-TG. Relative heart mass was increased in *Sgcg*^{TG} compared to *Sgcg*. (**D**) Overexpression of SSPN did not significantly reduce CK level in α-SG and β-SG deficient mice. In sarcoglycan deficient mice, mean plasma CK was elevated fourfold relative to wildtype mice. Mice aged 12-20 weeks (n = 7-18 per genotype) were analyzed. Data presented as mean ± standard deviation. Statistical analysis was performed by one-way ANOVA followed by Tukey's multiple comparison test.

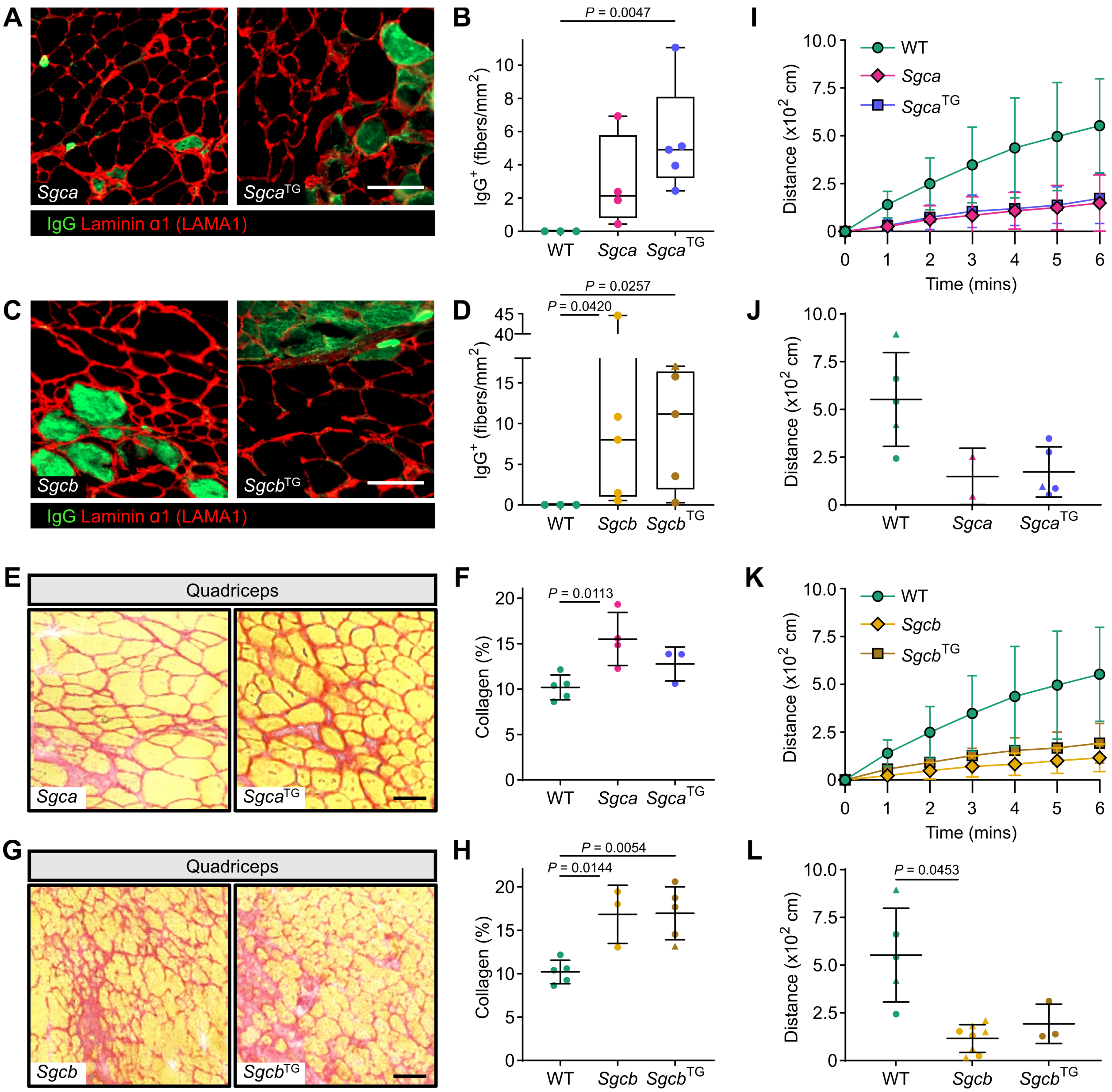

Supplemental Figure 3. SSPN does not ameliorate *Sgca* and *Sgcb* muscular dystrophy. (**A**) Representative images and (**B**) IgG+ fiber quantification in *Sgca* and *Sgca*^{TG} quadriceps cryosections. (**C**) Representative images and (**D**) IgG+ fiber quantification in *Sgcb* and *Sgcb*^{TG} quadriceps cryosections. IgG+ fiber density is not reduced in *Sgca*^{TG} and *Sgcb*^{TG} relative to *Sgca* and *Sgcb*, n=3-5 mice per genotype. Statistical analysis by Krusal-Wallis test and Conover-Iman test with Bonferroni correction. (**E**) Representative images and (**F**) Picrosirius red quantification of *Sgca* and *Sgca*^{TG} quadriceps cryosections. (**G**) Representative images and (**H**) Picrosirius red quantification of *Sgcb* and *Sgcb*^{TG} quadriceps cryosections. Collagen is not reduced in *Sgca*^{TG} and *Sgcb*^{TG} compared to *Sgca* and *Sgcb* muscle, n=3-6 mice per genotype. Statistical analysis by one-way ANOVA and Tukey's test. Bar, 100 μm (**I**) *Sgca* and *Sgca*^{TG} post-exercise ambulation distance and (**J**) total ambulation distance, n=2-5 per genotype. (**K**) *Sgcb* and *Sgcb*^{TG} post-exercise ambulation and (**L**) total ambulation distance, n=3-5 per genotype. Statistical analysis of total distance by Welch's ANOVA and Dunnett test. Circles, male mice; triangles, female mice.

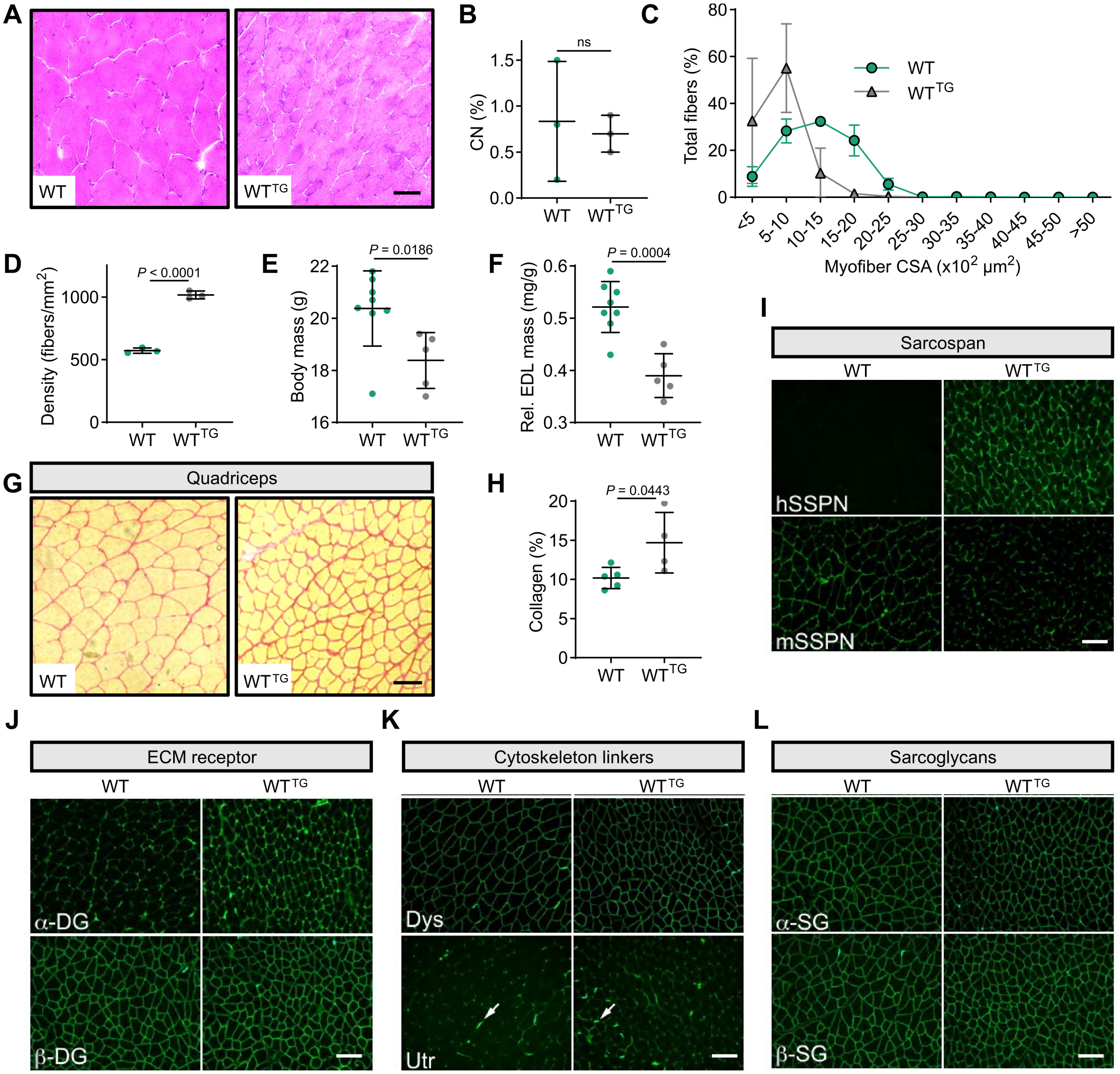

Supplemental Figure 4. SSPN decreases muscle size and body mass in wildtype mice. (**A**) H&E images of wildtype and wildtype SSPN-transgenic (WT^{TG}) muscle. Quantification of (**B**) central nucleation (CN), (**C**) muscle fiber CSA, and (**D**) muscle fiber density of wildtype and wildtype transgenic mice. Bar, 50 µm. (**E**) Body mass and (**F**) muscle mass (extensor digitorum longus relative to body mass) of wildtype and wildtype transgenic mice. SSPN reduces muscle fiber area, increases fiber density, and reduces body mass and muscle mass in wildtype mice. Statistical analysis by Welch's unpaired *t*-test (**B**); unpaired *t*-test (**D**) and (**F**); Wilcoxon-rank sum test (**E**). (**G**) Representative images and (**H**) quantification of Picrosirius red stained (collagen) wildtype and wildtype transgenic quadriceps showing that collagen is more abundant in transgenic muscle. Bar, 100 µm. Immunofluorescence images of wildtype and wildtype transgenic quadriceps muscle probed for (**I**) sarcospan, (**J**) alpha- and beta-dystroglycan (DG), (**K**) dystrophin (Dys) and utrophin (Utr), and (**L**) alpha- and beta-sarcoglycan (SG). Bar, 200 µm.

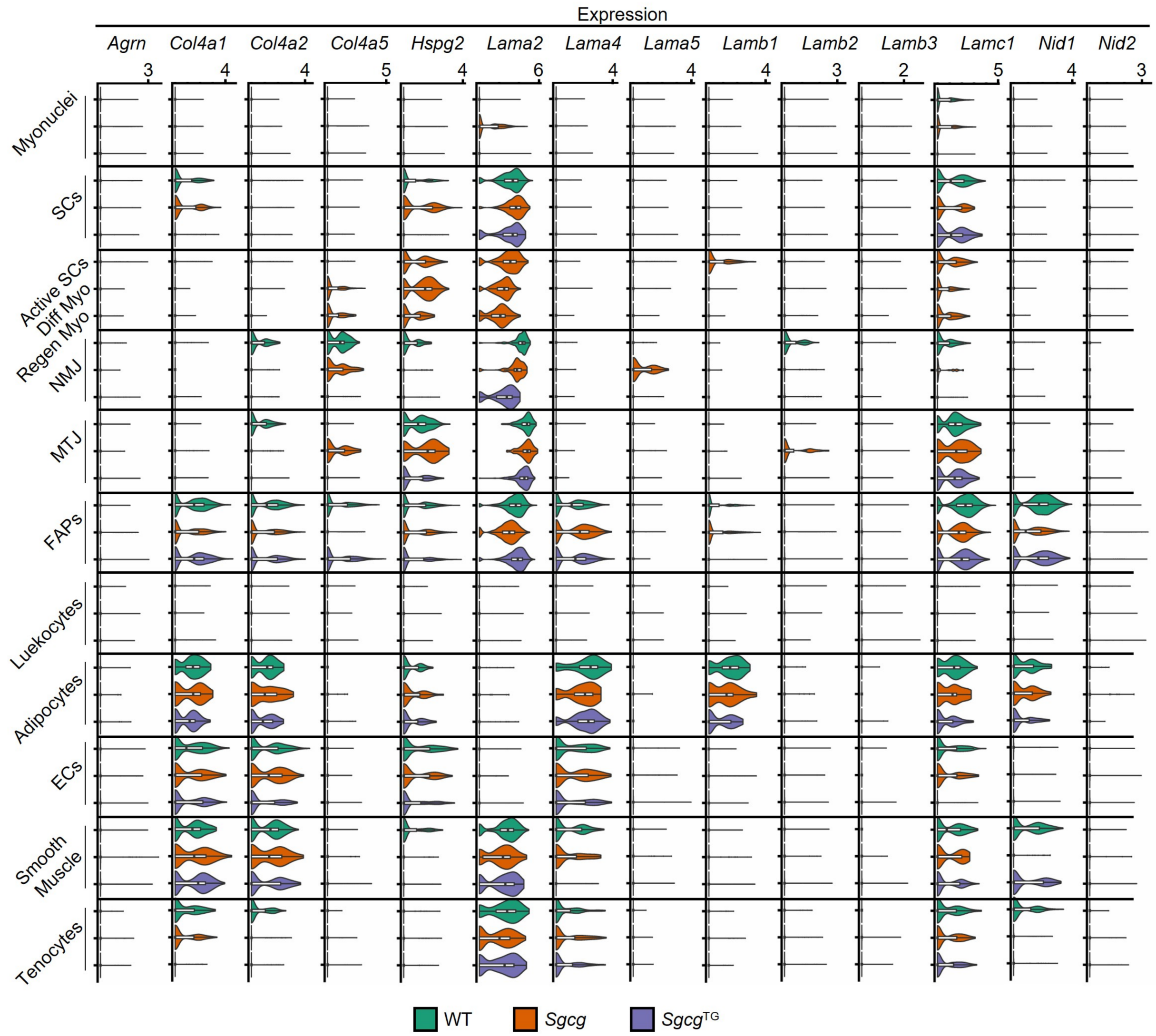

Supplemental Figure 5. Cell-type specific basement membrane expression patterns in skeletal muscle reveal decreased expression of the laminin-nidogen-perlecan-Col IV structural backbone in FAPs. Normalized gene expression values encoding fourteen basement membrane proteins (top row) from distinct cell populations (first column) identified in single nuclei RNA sequencing data from wildtype (green) *Sgcg* (orange), and *Sgcg*^{TG} (purple) quadriceps muscle. Basement membrane protein expression was increased in myogenic lineage cells (SCs, Diff Myo, Regen Myo) and decreased in FAPs from *Sgcg* quadriceps relative to wildtype FAPs. SCs, satellite cells; Diff Myo, differentiating myoblasts; Regen Myo, regenerating myonuclei; NMJ, neuromuscular junction; MTJ, myotendinous junction; FAPs, fibro-adipogenic progenitors; ECs, endothelial cells.

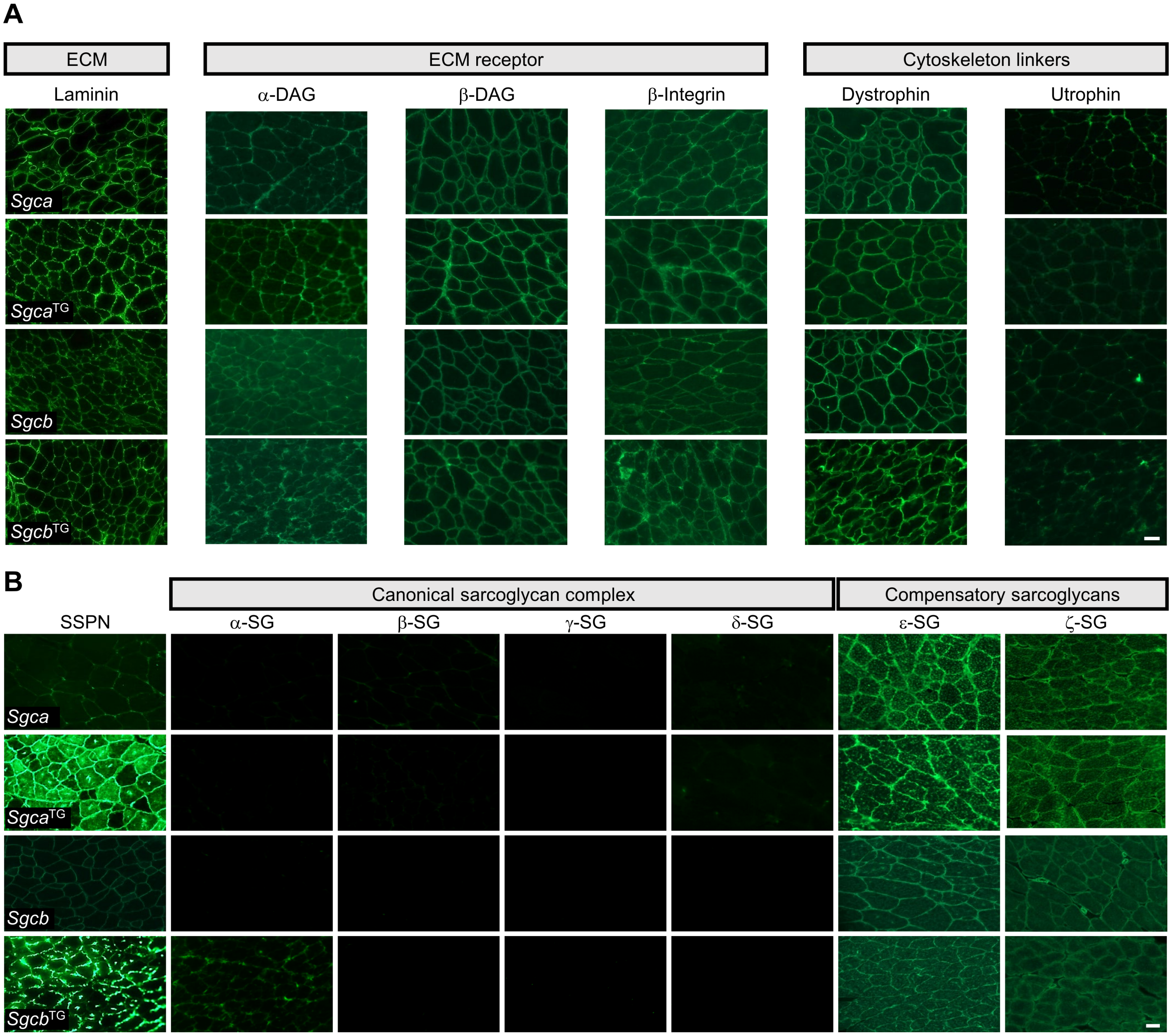

Supplemental Figure 6. SSPN fails to restore sarcoglycan membrane localization in *Sgca* and *Sgcb* skeletal muscle. (**A**) Indirect immunofluorescence assays of transverse *Sgca*, *Sgca*^{TG}, *Sgcb*, and *Sgcb*^{TG} quadriceps cryosections using antibodies to a marker of the basement membrane (laminin), laminin receptors (α-/β-dystroglycan and β1-integrin), and two cytoskeletal linkers (dystrophin and utrophin), as indicated. While β1-integrin was slightly increased in *Sgcb*^{TG} mice compared to *Sgcb*, all other proteins were unaffected by SSPN overexpression. Bar, 50 µm. (**B**) Indirect immunofluorescence assays of transverse *Sgca*, *Sgca*^{TG}, *Sgcb*, and *Sgcb*^{TG} quadriceps cryosections using antibodies to SSPN and the indicated SGs. Unlike *Sgcg* muscle, SSPN failed to restore membrane localization of the canonical SGs. SSPN did not alter expression of the non-canonical SGs. Bar, 50 µm.

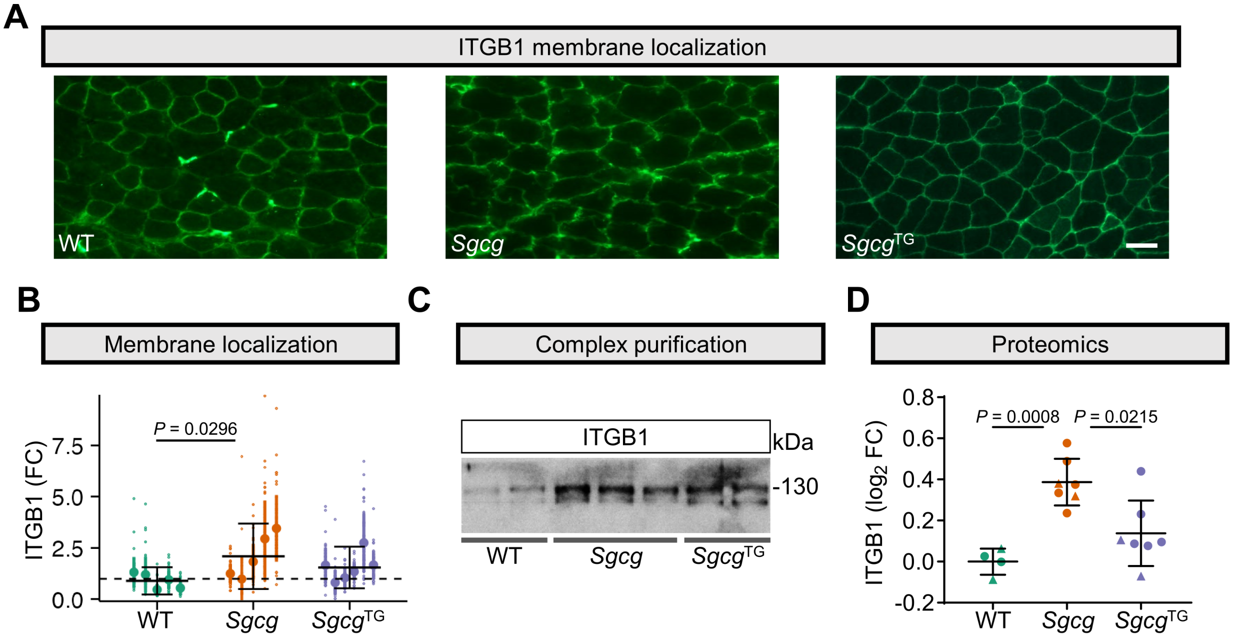

Supplemental Figure 7. Increased β1-integrin expression in *Sgcg* muscle associates with dystroglycan to compensate for DGC destabilization. (**A**) Indirect immunofluorescence assays of transverse wildtype, *Sgcg*, and *Sgcg*^{TG} quadriceps cryosections probed with β1-integrin antibodies. Bar, 50 µm. (**B**) Quantification of β1-integrin abundance at the sarcolemma, n = 5-6 per genotype, 49 individual myofiber measurements per mouse (total 245-294). Data are presented as fold change (FC) relative to wildtype (mean ± standard deviation) for individual mice within a genotype. The mean ± standard deviation for each genotype is provided with black solid bars (dashed line represents mean of wildtype samples). Statistical analysis was performed by fitting a generalized linear model. (**C**) Immunobloting of the eluate fractions of lectin (sWGA) purifications from all three genotypes using β1-integrin antibodies (n=2-3 per genotype). Proteins that interact in a complex can be detected in eluate fractions. β1-integrin robustly associates with dystroglycan in *Sgcg* and *Sgcg*^{TG} samples, but not wildtype. (**D**) Relative protein abundance from global proteomics analysis revealed increased integrin β1 in *Sgcg* compared to wildtype and reduced to near wildtype levels in *Sgcg*^{TG}. Statistical analysis for proteomics data conducted by unpaired *t*-tests and Benjamini-Hochberg procedure. Circles, male mice; triangles, female mice.

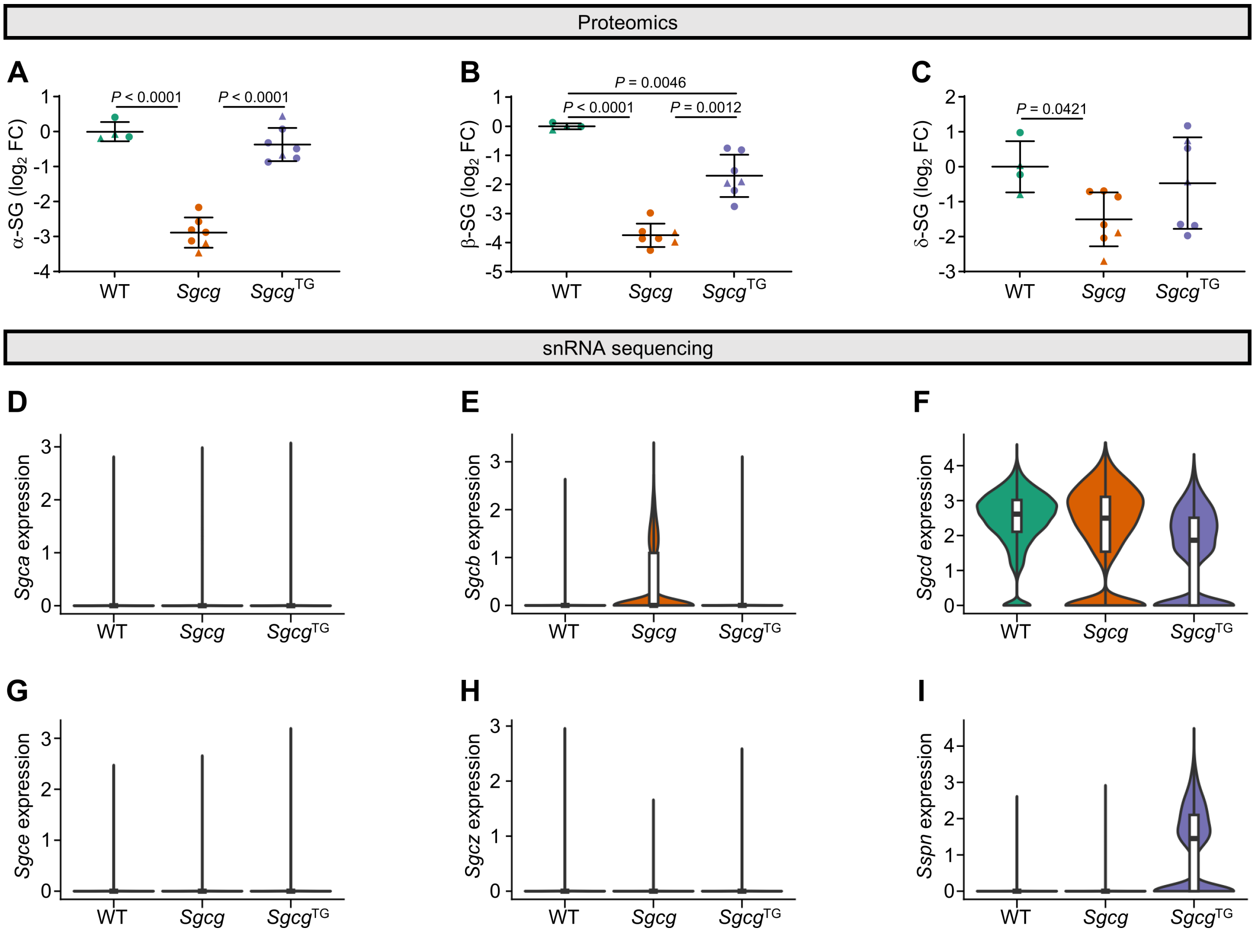

Supplemental Figure 8. SSPN increases total protein abundance of canonical sarcoglycans in *Sgcg* skeletal muscle without affecting their gene expression. (**A-C**) The absence of γ-sarcoglycan in skeletal muscle decreased total protein abundance of α-, β- and δ-sarcoglycan, while transgenic SSPN expression increased total protein levels of sarcoglycans in *Sgcg*^{TG} muscles (n = 4-7 per genotype). Circles, male mice; triangles, female mice. (**D-I**) Expression of sarcolgycans and SSPN in myonuclei from single nuclei RNA sequencing. Sarcoglycans, except *Sgcd*, were expressed at low levels across all genotypes. Violin plots show gene expression distributions in myonuclei. Boxplots overlay violin plots showing median, interquartile range, and minimum and maximum values. Despite subtle differences in expression, relative protein abundances of sarcoglycans are significantly different between genotypes. Differential protein expression conducted on entire dataset by pairwise two-sided *t*-tests followed by Benjamini–Hochberg procedure to control for false discovery rate.

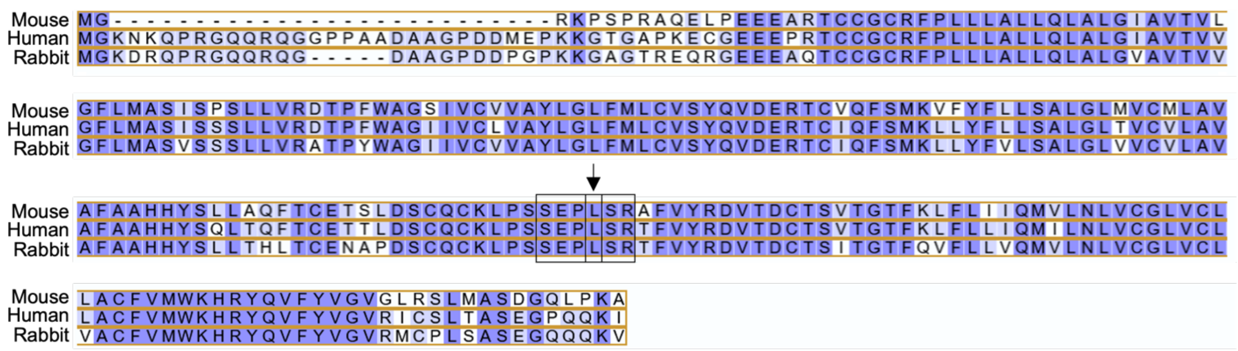

Supplemental Figure 9. SSPN extracellular loop Leu145 is conserved across species. Multiple Sequence alignment of murine (UniProt ID Q62147), rabbit (UniProt ID P82352), and human SSPN (UniProt Q14714) proteins. Note that murine Leu145 is conserved with human Leu172 and rabbit Leu167 (black arrow). The SEPLSR amino acid sequence is in the large extracellular loop of SSPN.

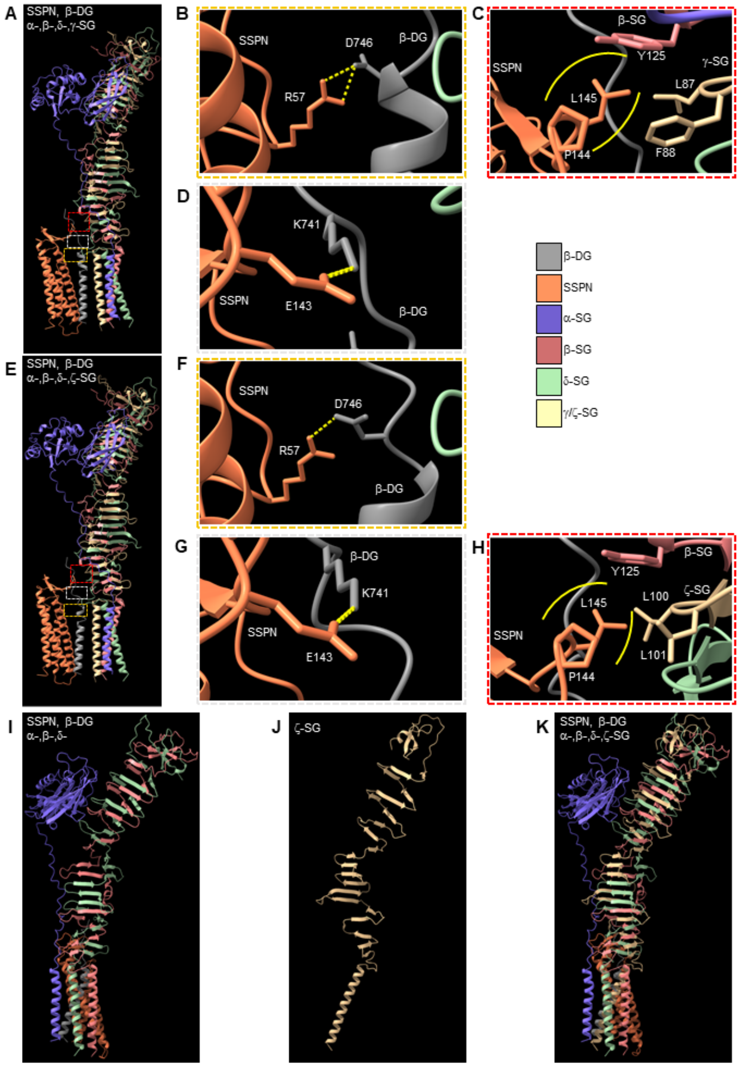

Supplemental Figure 10. Structure and interaction sites are conserved in the predicted ζ-SG and γ-SG containing β-DG-SSPN-SG complexes. AlphaFold 3 predicted structures of (**A**) β-DG (aa. 736-773) and SSPN associating with the α-, β-, δ-, γ-SG complex. (**B**) An inset (orange dashed box in (**A**)) showing the site of a salt bridge between Arg57 of SSPN and Asp746 of β-DG. (**C**) An inset (red dashed box in (**A**)) showing the hydrophobic interactions between Pro144 and Leu145 of SSPN, Leu87 and Phe88 of γ-SG, and Tyr125 of β-SG. (**D**) An inset (grey dashed box in (**A**)) showing the salt bridge formed between Glu143 of SSPN and Lys741 of β-DG. (**E**) β-DG (aa. 736-773) and SSPN associating with the α-, β-, δ-, ζ-SG complex demonstrates high similarity with the canonical γ-SG containing complex shown in (**A**). (**F**) An inset (orange dashed box in (**E**)) showing a conservation of the salt bridge shown in (**B**). (**G**) An inset (grey dashed box in (**E**)) showing conservation of the salt bridge shown in (**D**). (**H**) An inset (red dashed box in (**E**)) showing a conservation of the hydrophobic interactions shown in **(C)** with Leu100 and Leu101 of ζ-SG taking the place of Leu87 and Phe88 of γ-SG**.** Dotted yellow lines, hydrogen bonds involved in a

salt bridge; yellow arcs, hydrophobic interactions. (**I**) The complex without γ-SG, showing an open binding pocket that can be filled by (**J**) ζ-SG to form the (**K**) α-, β-, δ-, ζ-SG complex.

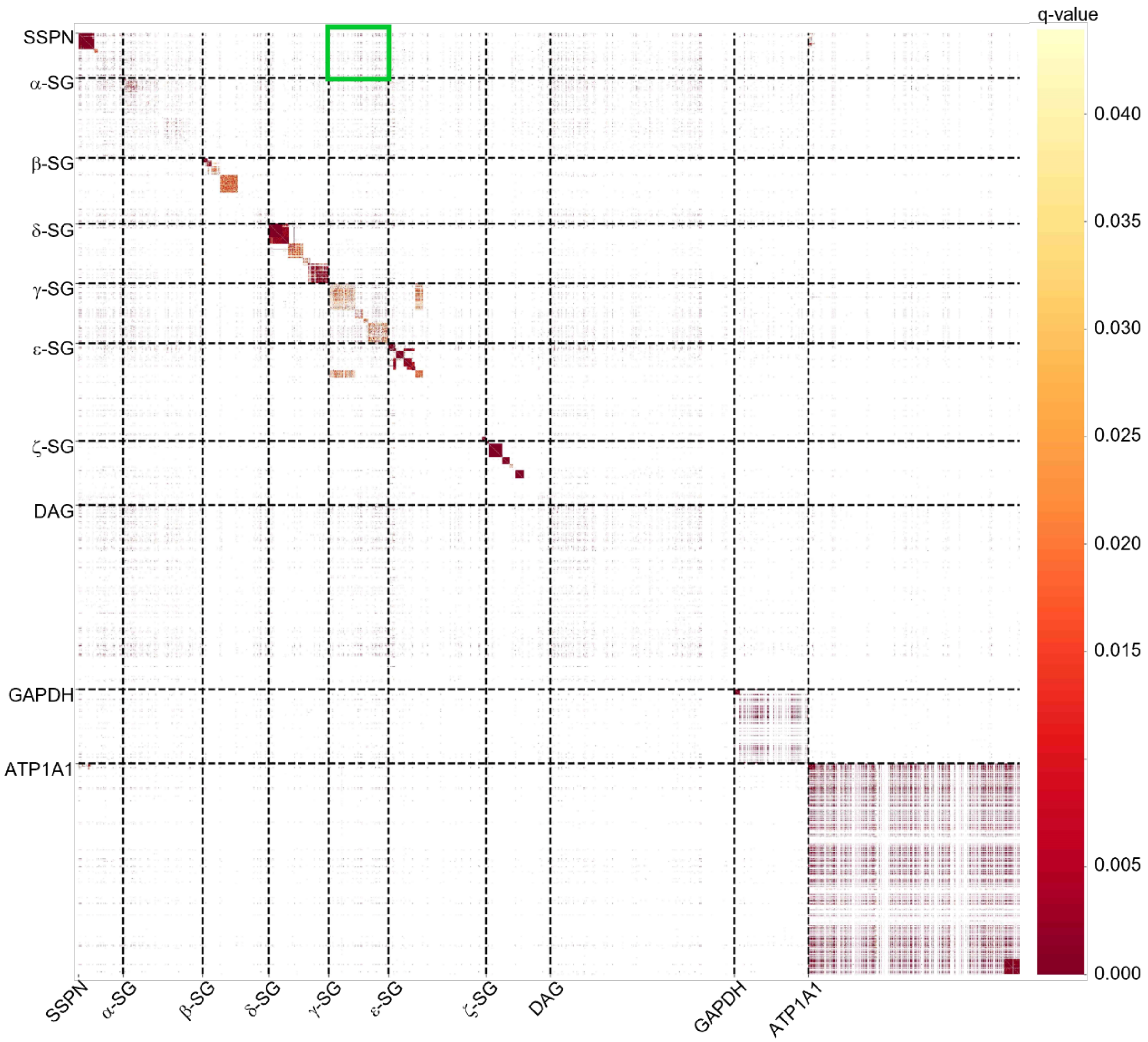

Supplemental Figure 11. Heatmap visualization of significant co-evolutionary relationships (*q*-value < 0.05) between protein residues. The diagonal represents intra-protein residue pairs, showing a higher proportion of significant co-evolution. Notably, the highest fraction of inter-protein co-evolving residue pairs was observed between SSPN and γ-SG (bordered with green). Note that residue numbers (not shown) are mapped to the corresponding residue numbers in the mouse protein sequence as a reference.

Supplementary Table 1. Quantitative proteomics reveals dysregulated deposition of the core basement membrane proteins in early stage *Sgcg* muscle.

Protein	WT (%)	*Sgcg* (%)	*Sgcg*^{TG} (%)
**AGRN**	7.7±0.4	5.1±1.3*	6.7±1.0
**COL4A1**	12.8±0.0	11.5±1.5	14.1±1.2$^{\#}$
**COL4A1/5**	11.0±0.8	9.7±0.9	12.0±1.8$^{\#}$
**COL4A2**	4.9±0.3	4.7±0.4	5.8±0.3$^{\#}$
**Canstatin**	7.4±0.5	6.6±0.8	8.1±0.3$^{\#}$
**COL4A5**	3.3±0.4	8.1±4.5	3.4±0.9
**HSPG2**	16.8±0.4	17.1±1.7	15.6±1.0
**Endorepellin**	12.2±0.3	12.4±1.1	11.5±0.7
**LAMA2**	2.3±0.2	2.0±0.2	2.1±0.1
**LAMA4**	1.2±0.2	1.4±0.1	1.1±0.2$^{\#}$
**LAMA5**	0.7±0.1	0.5±0.1	0.5±0.1
**LAMB1**	1.7±0.2	1.9±0.2	1.9±0.1
**LAMB2**	1.8±0.1	1.7±0.2	1.8±0.2
**LAMB3**	2.3±0.3	3.6±1.4	2.1±0.4$^{\#}$
**LAMC1**	2.4±0.2	2.4±0.2	2.3±0.1
**NID1**	2.7±0.4	2.9±0.4	2.8±0.2
**NID1/2**	6.3±0.8	6.6±0.5	6.1±0.4
**NID2**	2.7±0.3	1.7±0.4*	2.1±0.4

Nidogen, perlecan, laminin, and Col IV form the core structural backbone of the basement membrane. Values from data presented in Figure 5B. The absolute quantity of basement membrane proteins was determined by mass spectrometry using quantitative concatemers (QconCATs) (17, 18). Each basement membrane protein is represented as a percentage of the total basement membrane to assess basement membrane compositional shift. Values show mean ± standard deviation. The matrikines canstatin and endorepellin produced from peptide sequences found in the C-terminus of COL4A2 and HSPG2 proteins, respectively. NID1/2 represents the peptide sequence GNLYWTDWNR found in both NID1 and NID2. COL4A1/5 represents the peptide sequence SAPFIECHGR found in both COL4A1 and COL4A5. Statistical analysis conducted by Kruskal-Wallis test and Dunn's test. *Statistically different from wildtype; $^{\#}$statistically different from *Sgcg*.

Supplementary Table 2. Summary of quantitative proteomic analysis of absolute concentration and solubility of basement membrane proteins.

	Absolute Quantity (nmol/g)			ECM Solubility (%)		
**Protein**	**WT**	***Sgcg***	***Sgcg*^{TG}**	**WT**	***Sgcg***	***Sgcg*^{TG}**
**AGRN**	0.46±0.05	0.43±0.05	0.52±0.05$^{\#}$	82.4±7.6	85.8±3.9	84.0±4.7
**COL4A1**	0.76±0.05	0.99±0.15	1.11±0.19*	73.6±4.7	75.1±3.4	77.0±3.8
**COL4A1/5**	0.66±0.08	0.84±0.15	0.95±0.19*	72.7±6.4	78.9±3.2	76.59±6.2
**COL4A2**	0.29±0.04	0.40±0.06*	0.46±0.05*	53.0±3.4	55.1±8.9	60.09±6.5
**Canstatin**	0.44±0.04	0.57±0.07*	0.64±0.07*	67.9±1.7	62.7±6.1	70.89±4.6$^{\#}$
**COL4A5**	0.20±0.04	0.75±0.55*	0.27±0.06	N/A	N/A	N/A
**HSPG2**	1.00±0.09	1.48±0.24*	1.24±0.18	86.2±2.7	86.6±3.8	86.11±3.0
**Endorepellin**	0.72±0.06	1.08±0.17*	0.91±0.13	86.0±2.5	86.5±3.4	85.9±2.9
**LAMA2**	0.13±0.01	0.17±0.01*	0.17±0.02*	82.4±2.3	83.9±2.2	79.6±2.6$^{\#}$
**LAMA4**	0.07±0.01	0.12±0.02*	0.09±0.01‡	N/A	N/A	N/A
**LAMA5**	0.04±0.01	0.05±0.01	0.04±0.01	70.6±12.7	69.7±4.6	65.5±9.9
**LAMB1**	0.10±0.00	0.17±0.02*	0.15±0.02*	80.4±3.4	82.8±4.9	78.5±4.7
**LAMB2**	0.11±0.01	0.15±0.01*	0.14±0.02*	82.2±1.3	84.2±2.5	83.3±2.9
**LAMB3**	0.14±0.02	0.33±0.17*	0.17±0.03	75.3±15.2	87.3±7.0	79.2±7.4
**LAMC1**	0.14±0.01	0.21±0.02*	0.18±0.02‡	84.6±1.2	86.0±2.7	83.3±3.5
**NID1**	0.16±0.02	0.25±0.02*	0.22±0.02*	89.3±2.2	89.8±3.0	89.4±2.0
**NID1/2**	0.37±0.02	0.57±0.06*	0.48±0.04‡	91.4±2.9	90.1±1.3	91.1±1.5
**NID2**	0.16±0.02	0.15±0.02	0.16±0.02	84.5±2.9	81.2±5.3	84.3±4.2

The absolute quantity of basement membrane proteins determined by mass spectrometry and quantitative concatemers (17, 18). Absolute protein quantity values summarized from Fig. 5 and 6. Samples were fractioned based on solubility by chemical denaturation and absolute quantities of proteins were determined by mass spectrometry. Briefly, proteins were extracted into fractions and defined as cellular, soluble extracellular matrix (sECM), or insoluble extracellular matrix (iECM). The proportion of protein quantified in the sECM fraction compared to the total ECM was defined as percent solubility (solubility (%) $= \frac{\text{sECM}}{\text{sECM+iECM}} \times 100$). Data represent mean ± standard deviation. The matrikines canstatin and endorepellin are peptide sequences found in the C-terminus of COL4A2 and HSPG2 proteins, respectively. NID1/2 represents the peptide sequence GNLYWTDWNR found in both NID1 and NID2. COL4A1/5 represents the peptide sequence SAPFIECHGR found in both COL4A1 and COL4A5. Protein concentrations, excluding COL4A5, LAMA4, LAMB3, were compared using one-way ANOVA with Tukey's multiple comparisons test. COL4A5 and LAMB3 were compared using Kruskal-Wallis test followed by Dunn's test. LAMA4 concentrations were compared using Welch's ANOVA and Dunnett test. One-way ANOVA and Tukey's test was used to compare solubility, except for canstatin, HSPG2, endorepellin, LAMA2, LAMB3, and NID1/2. Solubility for canstatin, HSPG2, endorepellin, and LAMA2 was compared by Kruskal-Wallis test followed by Dunn's test. LAMB3 and NID1/2 solubility were compared using Welch's ANOVA and Dunnett test. *Statistically different from wildtype; $^{\#}$statistically different from *Sgcg*; ‡statistically different from wildtype and *Sgcg*; N/A, no protein detected in either sECM or iECM, making solubility calculation invalid.

Supplemental Table 3. Mammalian multiple sequence alignment reveals binding site conservation between SSPN and γ-sarcoglycan or ζ-sarcoglycan.

Protein	Murine AA site	Identical Residue Conserved	Similar Residue Conserved
γ-SG	Phe88	97%	100%
ζ-SG	Leu101	100%	100%
SSPN	Leu145	98%	100%

Multiple sequence alignments showed that binding site residues between SSPN and either γ-sarcoglycan or ζ-sarcoglycan are highly conserved in both identity and similarity. This demonstrates necessity of these binding sites in SSPN-SG interaction and support exchangeability of γ-sarcoglycan or ζ-sarcoglycan in the SSPN-SG complex. AA, amino acid.

Supplementary Table 4. Top 20 co-evolving residue pairs ranked by mutual information (MI) score in descending order.

Protein1	Residue1	IC Score1	Protein2	Residue2	IC Score2	MI Score
**SSPN**	Pro163	1.82	**DAG**	Asn70	2.32	1.06
**SSPN**	Leu162	2.40	**DAG**	His74	2.58	1.06
**γ-SG**	Val312	2.10	**GAPDH**	Cys136	2.05	1.05
**γ-SG**	His78	1.55	**DAG**	Asn70	2.32	1.03
**γ-SG**	Ala131	1.94	**DAG**	Asn70	2.32	1.02
**γ-SG**	Val65	2.35	**DAG**	Asn70	2.32	1.02
**α-SG**	Leu491	2.50	**DAG**	Trp71	2.30	1.01
**SSPN**	Pro150	2.04	**γ-SG**	Asp233	2.15	1.01
**γ-SG**	Val104	2.96	**ε-SG**	Asp472	3.25	1.00
**SSPN**	Pro157	2.02	**γ-SG**	Lys326	2.04	0.99
**SSPN**	Leu162	2.40	**DAG**	Gly800	2.67	0.98
**α-SG**	Val282	1.92	**DAG**	Asn70	2.32	0.98
**SSPN**	Pro150	2.04	**γ-SG**	Lys326	2.04	0.98
**SSPN**	Pro150	2.04	**γ-SG**	Val312	2.10	0.97
**SSPN**	Pro163	1.82	**γ-SG**	His78	1.55	0.96
**α-SG**	Arg292	2.59	**γ-SG**	Arg284	2.70	0.95
**α-SG**	Thr176	1.64	**γ-SG**	His78	1.55	0.95
**α-SG**	Arg244	2.82	**DAG**	His74	2.58	0.94

To ensure co-evolution was not constrained by individual conservation, information content (IC) was calculated for each residue (maximum score 4.2). The top co-evolving pairs demonstrate moderate conservation, avoiding highly conserved regions and allowing for variability necessary for mutual information analysis. Five of these top pairs are between SSPN and γ-SG, highlighting their strong co-evolutionary relationship while accounting for conservation bias. Note that residue numbers refer to the specific residue in the corresponding mouse protein sequence.

REFERENCES

1. Durbeej M, Cohn RD, Hrstka RF, Moore SA, Allamand V, Davidson BL, et al. Disruption of the beta-sarcoglycan gene reveals pathogenetic complexity of limb-girdle muscular dystrophy type 2E. *Mol Cell.* 2000;5(1):141-51.
2. Hack AA, Ly CT, Jiang F, Clendenin CJ, Sigrist KS, Wollmann RL, et al. Gamma-sarcoglycan deficiency leads to muscle membrane defects and apoptosis independent of dystrophin. *J Cell Biol.* 1998;142(5):1279-87.
3. Marshall JL, Holmberg J, Chou E, Ocampo AC, Oh J, Lee J, et al. Sarcospan-dependent Akt activation is required for utrophin expression and muscle regeneration. *J Cell Biol.* 2012;197(7):1009-27.
4. Peter AK, Marshall JL, and Crosbie RH. Sarcospan reduces dystrophic pathology: stabilization of the utrophin-glycoprotein complex. *J Cell Biol.* 2008;183(3):419-27.
5. Gibbs EM, Marshall JL, Ma E, Nguyen TM, Hong G, Lam JS, et al. High levels of sarcospan are well tolerated and act as a sarcolemmal stabilizer to address skeletal muscle and pulmonary dysfunction in DMD. *Hum Mol Genet.* 2016;25(24):5395-406.
6. Mamsa H, Stark RL, Shin KM, Beedle AM, and Crosbie RH. Sarcospan increases laminin-binding capacity of alpha-dystroglycan to ameliorate DMD independent of Galgt2. *Hum Mol Genet.* 2022;31(5):718-32.
7. Seo YE, Baine SH, Kempton AN, Rogers OC, Lewis S, Adegboye K, et al. Systemic γ-sarcoglycan AAV gene transfer results in dose-dependent correction of muscle deficits in the LGMD 2C/R5 mouse model. *Mol Ther Methods Clin Dev.* 2023;28:284-99.
8. Boyer JG, Huo J, Han S, Havens JR, Prasad V, Lin BL, et al. Depletion of skeletal muscle satellite cells attenuates pathology in muscular dystrophy. *Nat Commun.* 2022;13(1):2940.
9. Faul F, Erdfelder E, Buchner A, and Lang A-G. Statistical power analyses using G*Power 3.1: Tests for correlation and regression analyses. *Behavior Research Methods.* 2009;41(4):1149-60.
10. Thompson JD, Higgins DG, and Gibson TJ. CLUSTAL W: improving the sensitivity of progressive multiple sequence alignment through sequence weighting, position-specific gap penalties and weight matrix choice. *Nucleic Acids Res.* 1994;22(22):4673-80.
11. Gloor GB, Martin LC, Wahl LM, and Dunn SD. Mutual information in protein multiple sequence alignments reveals two classes of coevolving positions. *Biochemistry.* 2005;44(19):7156-65.
12. Bankhead P, Loughrey MB, Fernández JA, Dombrowski Y, McArt DG, Dunne PD, et al. QuPath: Open source software for digital pathology image analysis. *Sci Rep.* 2017;7(1):16878.
13. Peter AK, and Crosbie RH. Hypertrophic response of Duchenne and limb-girdle muscular dystrophies is associated with activation of Akt pathway. *Exp Cell Res.* 2006;312(13):2580-91.
14. Flores I, Welc SS, Wehling-Henricks M, and Tidball JG. Myeloid cell-mediated targeting of LIF to dystrophic muscle causes transient increases in muscle fiber lesions by disrupting the recruitment and dispersion of macrophages in muscle. *Hum Mol Genet.* 2021;31(2):189-206.
15. Landini G, Martinelli G, and Piccinini F. Colour deconvolution: stain unmixing in histological imaging. *Bioinformatics.* 2021;37(10):1485-7.
16. Gibbs EM, and Crosbie-Watson RH. A Simple and Low-cost Assay for Measuring Ambulation in Mouse Models of Muscular Dystrophy. *J Vis Exp.* 2017(130).
17. Beynon RJ, Doherty MK, Pratt JM, and Gaskell SJ. Multiplexed absolute quantification in proteomics using artificial QCAT proteins of concatenated signature peptides. *Nat Methods.* 2005;2(8):587-9.
18. Pratt JM, Simpson DM, Doherty MK, Rivers J, Gaskell SJ, and Beynon RJ. Multiplexed absolute quantification for proteomics using concatenated signature peptides encoded by QconCAT genes. *Nat Protoc.* 2006;1(2):1029-43.
19. Johnson TD, Hill RC, Dzieciatkowska M, Nigam V, Behfar A, Christman KL, et al. Quantification of decellularized human myocardial matrix: A comparison of six patients. *Proteomics Clin Appl.* 2016;10(1):75-83.
20. Wiśniewski JR, Zougman A, Nagaraj N, and Mann M. Universal sample preparation method for proteome analysis. *Nat Methods.* 2009;6(5):359-62.
21. Zhang X, Smits AH, van Tilburg GB, Ovaa H, Huber W, and Vermeulen M. Proteome-wide identification of ubiquitin interactions using UbIA-MS. *Nat Protoc.* 2018;13(3):530-50.

22. Huber W, von Heydebreck A, Sültmann H, Poustka A, and Vingron M. Variance stabilization applied to microarray data calibration and to the quantification of differential expression. *Bioinformatics.* 2002;18 Suppl 1:S96-104.
23. Gatto L, Gibb S, and Rainer J. MSnbase, Efficient and Elegant R-Based Processing and Visualization of Raw Mass Spectrometry Data. *J Proteome Res.* 2021;20(1):1063-9.
24. Välikangas T, Suomi T, and Elo LL. A systematic evaluation of normalization methods in quantitative label-free proteomics. *Brief Bioinform.* 2018;19(1):1-11.
25. Hill RC, Calle EA, Dzieciatkowska M, Niklason LE, and Hansen KC. Quantification of extracellular matrix proteins from a rat lung scaffold to provide a molecular readout for tissue engineering. *Mol Cell Proteomics.* 2015;14(4):961-73.
26. MacLean B, Tomazela DM, Shulman N, Chambers M, Finney GL, Frewen B, et al. Skyline: an open source document editor for creating and analyzing targeted proteomics experiments. *Bioinformatics.* 2010;26(7):966-8.
27. Goddard ET, Hill RC, Barrett A, Betts C, Guo Q, Maller O, et al. Quantitative extracellular matrix proteomics to study mammary and liver tissue microenvironments. *Int J Biochem Cell Biol.* 2016;81(Pt A):223-32.
28. Hao Y, Stuart T, Kowalski MH, Choudhary S, Hoffman P, Hartman A, et al. Dictionary learning for integrative, multimodal and scalable single-cell analysis. *Nat Biotechnol.* 2024;42(2):293-304.
29. Traag VA, Waltman L, and van Eck NJ. From Louvain to Leiden: guaranteeing well-connected communities. *Sci Rep.* 2019;9(1):5233.
30. Petrany MJ, Swoboda CO, Sun C, Chetal K, Chen X, Weirauch MT, et al. Single-nucleus RNA-seq identifies transcriptional heterogeneity in multinucleated skeletal myofibers. *Nat Commun.* 2020;11(1):6374.
31. Liu S, Su T, Xia X, and Zhou ZH. Native DGC structure rationalizes muscular dystrophy-causing mutations. *Nature.* 2024.
