# Supplementary material for: Sarcospan protects against LGMD R5 via remodeling of the sarcoglycan complex composition in dystrophic mice
Source: J Clin Invest. 2025 Jun 19;135(17):e187868. doi: 10.1172/JCI187868 (PMC12404760; doi:10.1172/JCI187868)
Supplement: Unedited blot and gel images [file jci-135-187868-s270.pdf]

Figure 7

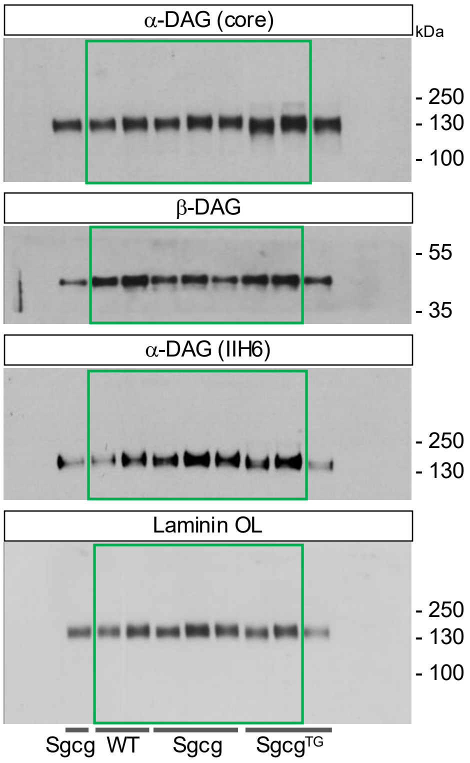

Figure 10

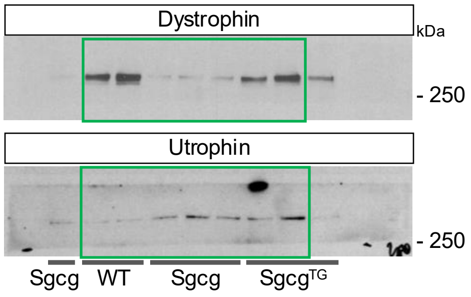

Supplemental Figure 7

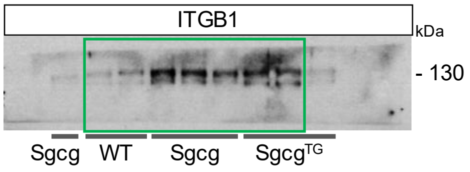

Figure 8

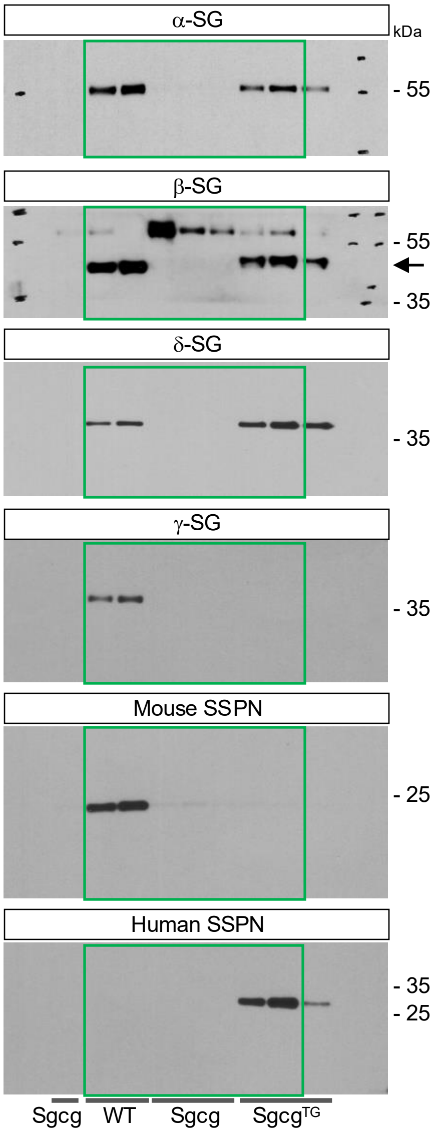

Figure 9

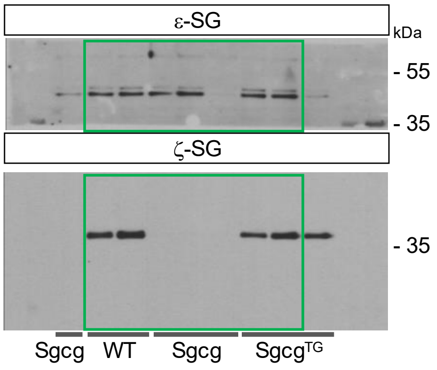

Uncropped Immunoblots of eluates from sWGA lectin purifications. Green box denotes samples included in the manuscript. The first and last samples on the immunoblots were excluded due to technical error.

Updated Laminin Overlay Blot 202050523

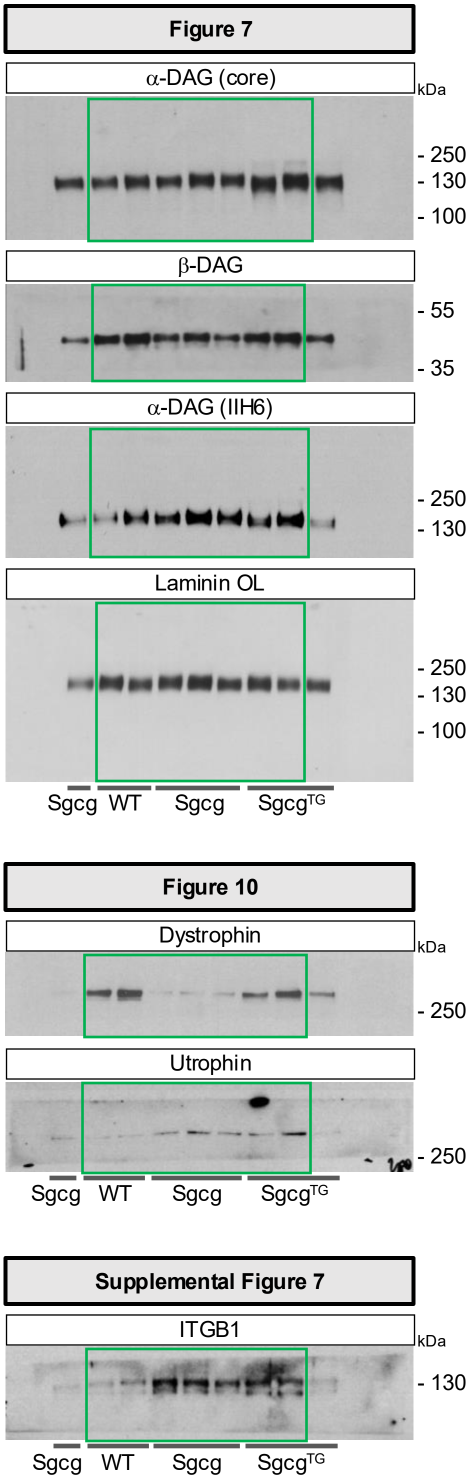

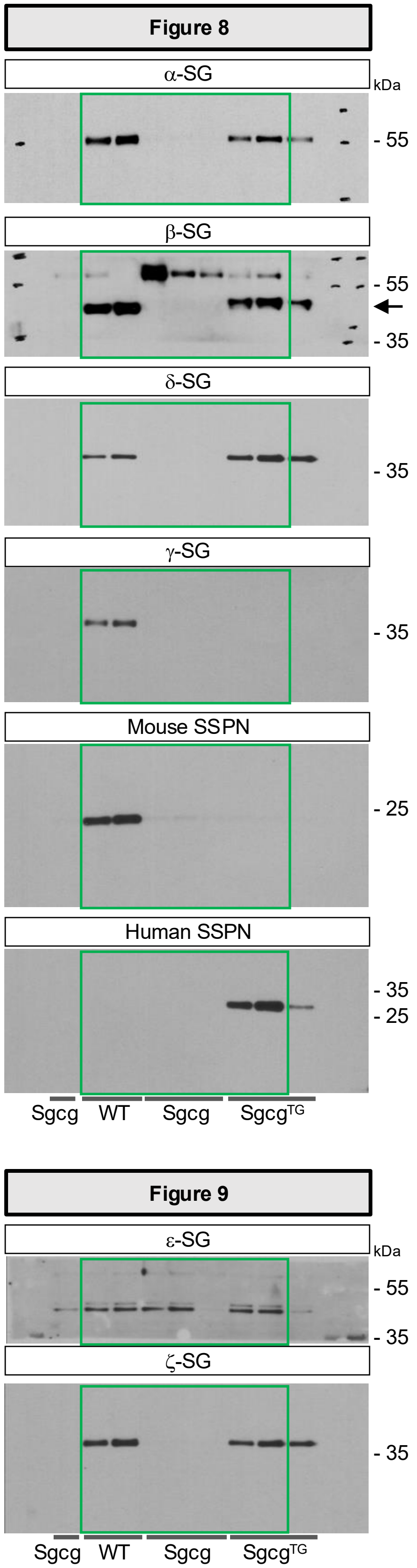

Uncropped Immunoblots of eluates from sWGA lectin purifications. Green box denotes samples included in the manuscript. The first and last samples on the immunoblots were excluded due to technical error.

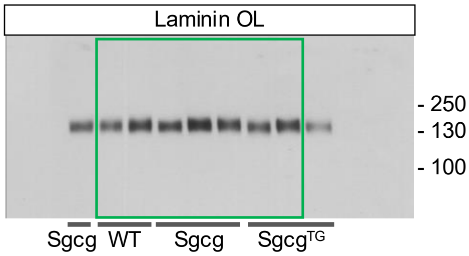

Original Submission

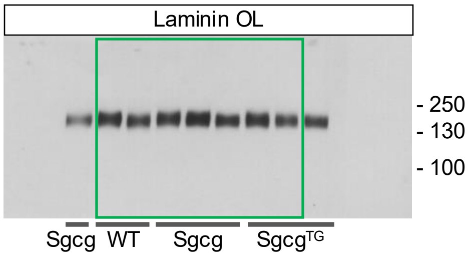

Re-submission 20250523
